# β-carboline alkaloids from *Trigonostemon filipes* and *Trigonostemon lii*

**DOI:** 10.1007/s13659-012-0028-x

**Published:** 2012-05-03

**Authors:** Shi-Fei Li, Yuan-Yuan Cheng, Yu Zhang, Shun-Lin Li, Hong-Ping He, Xiao-Jiang Hao

**Affiliations:** 1 State Key Laboratory of Phytochemistry and Plant Resources in West China, Kunming Institute of Botany, Chinese Academy of Sciences, Kunming, 650201 China; 2 Graduate University of Chinese Academy of Sciences, Beijing, 100049 China

**Keywords:** *Trigonostemon filipes*, *Trigonostemon lii*, *β*-carboline alkaloids, trifilines A-C, trigonoine C

## Abstract

Six *β*-carboline alkaloids including three new ones, trifilines A-C (**1**–**3**) were isolated from *Trigonostemon filipes*, along with a new *β*-carboline alkaloid, trigonoine C (**7**) isolated from *Trigonostemon lii*. Their structures were elucidated by extensive spectroscopic techniques. Trifilines A (**1**) and B (**2**) showed weak anti-HIV-1 activity with EC_50_ values of 54.61 µg/mL and 9.75 µg/mL, along with TI (Therapeutic index) values of 1.52 and 1.42, respectively. This is the first time to report the chemical constituents of *Trigonostemon filipes*.

**Electronic Supplementary Material:**

Supplementary material is available for this article at 10.1007/s13659-012-0028-x and is accessible for authorized users.

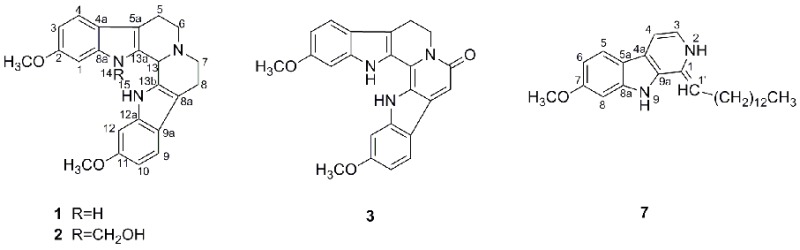

## Introduction

The genus *Trigonostemon* Bl. comprising about 50 species mainly is distributed in the tropical and subtropical regions of Asia, with 10 species being endemic to South China.[1] Previous chemical constituents and bioactivity investigations on this genus by our research group have involved an array of structurally interesting compounds such as modified daphnane-type diterpenoids, and diterpenoids, phenanthrenes, and indole alkaloids with anti-HIV-1 and cytotoxicity activity.[2] *T. filipes* is undershrub distributed in the Karst Eco Environment of Guangxi province.[1] So far, there has been no report on the chemical constitute from this species. In our further search for structurally interesting and bioactive compounds on this genus, a phytochemical work on *T. filipes* was conducted. In this work, six *β*-carboline alkaloids including three new ones, trifilines A–C (**1**–**3**), were isolated from a MeOH extract of *T. filipes*. Moreover, a new *β*-carboline alkaloids trigonoine C isolated from *T. lii* was reported herein. Trifilines A (**1**) and B (**2**) were tested for the anti-HIV-1 activities in preventing the cytopathic effects of HIV-$1_{IIIB}$ in C8166. Both of them showed weak anti-HIV-1 activity with $EC_{50}$ values of 54.61 *μ*g/mL and 9.75 *μ*g/mL, along with TI (Therapeutic index) values of 1.52 and 1.42, respectively.

## Results and Discussion

Trifiline A (**1**) was isolated as yellow amorphous solid. Its molecular formula was established as $C_{23}H_{23}N_3O_2$ on the basis

**1** R=H
**2** R=CH2OH
**3**
**4**
**5** R=H
**6** R=CH3
**7**

of a quasi-molecular ion peak at *m/z* 374.1875 [M + H]$^+$ in the HRESI mass spectrum. Its UV absorptions at 246, 271, and 301 nm suggested the existence of a *β*-carboline chromophore.[3] IR absorptions at 3349, 2899, 2840, 1631, 1499, and 1475 $cm^{-1}$ indicated the presence of NH, $CH_2$, and aromatic functionalities, respectively. The $^1H$ NMR spectrum (Table 1) showed proton signals attributed to six aromatic hydrogens, two methoxys [$\delta_H$ 3.74 (3H, s) and 3.73 (3H, s)] and two broad NH singlets [$\delta_H$ 10.63 (1H, br. s) and 10.72 (1H, br. s)]. The $^{13}C$ NMR spectrum accounted for all 23 carbon resonances comprising two methoxys, four $sp^3$ methylenes, seven methines (including six aromatic methines), and ten $sp^2$ quaternary carbons. Two AMX spin system ($\delta_H$ 6.61, 1H, dd, $J$ = 8.9, 2.8 Hz; 6.63, 1H, dd, $J$ = 8.9, 2.8 Hz; 6.83, 2H, d, $J$ = 2.8 Hz; 7.24, 1H, d, $J$ = 8.9 Hz; and 7.26, 1H, d, $J$ = 8.9 Hz) were observed on the basis of analysis NMR data as well as HSQC spectrum. In the HMBC spectrum, two NH singlets showed correlations to C-1a, C-4a, C-5a, and C-13a, and C-8a, C-9a, C-12a, and C-13b, respectively, suggesting that two indole units were determined. In addition, two identical partial

*To whom correspondence should be addressed. E-mail: haoxj@mail.kib.ac.cn (X.J. Hao); hehongping@mail.kib.ac.cn (H.P. He).

**Table 1. NMR data for trifilines A–C (1–3) in DMSO-$d_6$ ($\delta$ in ppm, $J$ in Hz)**

| No. | **1**[a] | | **2**[a] | | **3**[b] | |
|---|---|---|---|---|---|---|
| | $\delta_H$ | $\delta_C$ | $\delta_H$ | $\delta_C$ | $\delta_H$ | $\delta_C$ |
| 1 | 6.83, d (2.8) | 94.9, CH | 7.09, br. s | 94.2, CH | 6.89, d (2.2) | 94.5, CH |
| 1a | | 136.8, C | | 137.0, C | | 138.7, C |
| 2 | | 155.2, C | | 155.4, C | | 156.8, C |
| 3 | 6.63, dd (8.9, 2.8) | 108.1, CH | 6.72, dd (8.5, 1.8) | 108.6, CH | 6.71, dd (8.6, 2.2) | 109.6, CH |
| 4 | 7.26, d (8.9) | 117.8, CH | 7.29, d (8.5) | 117.8, CH | 7.44, d (8.6) | 120.1, CH |
| 4a | | 121.2, C | | 121.1, C | | 120.1, C |
| 5 | 3.32, dd (14.6, 3.2); 2.50, m | 27.6, $CH_2$ | 3.37, overlap; 2.52, overlap | 27.5, $CH_2$ | 3.02, t (6.7) | 19.4, $CH_2$ |
| 5a | | 106.1, C | | 106.4, C | | 110.5, C |
| 6 | 4.00, d (14.6); 3.60, m | 52.1, $CH_2$ | 4.22, d (14.8); 3.67, d (14.4) | 50.8, $CH_2$ | 4.43, t (6.7) | 40.2, $CH_2$ |
| 7 | 3.20, dd (9.8, 4.6); 2.69, dd (12.2, 9.8) | 52.5, $CH_2$ | 3.24, dd (9.8, 4.6); 2.75, overlap | 52.5, $CH_2$ | | 154.6, C |
| 8 | 2.79, m; 2.64, d (12.2) | 21.6, $CH_2$ | 2.82, overlap; 2.69, overlap | 21.6, $CH_2$ | 7.32, br. s | 93.7, CH |
| 8a | | 106.5, C | | 106.4, C | | 123.0, C |
| 9 | 7.24, d (8.9) | 118.3, CH | 7.29, d (8.5) | 118.2, CH | 7.81, d (8.7) | 121.8, CH |
| 9a | | 121.1, C | | 121.1, C | | 115.8, C |
| 10 | 6.61, dd (8.9, 2.8) | 108.1, CH | 6.65, dd (8.5, 1.8) | 108.0, CH | 6.85, dd (8.7, 2.2) | 110.8, CH |
| 11 | | 155.3, C | | 155.2, C | | 159.2, C |
| 12 | 6.83, d (2.8) | 94.9, CH | 6.86, d (1.8) | 94.7, CH | 6.95, d (2.2) | 94.5, CH |
| 12a | | 137.1, C | | 136.9, C | | 140.9, C |
| 13 | 3.63, br. s | 56.8, CH | 3.65, br. s | 56.8, CH | | 129.6, C |
| 13a | | 134.7, C | | 134.3, C | | 128.4, C |
| 13b | | 131.3, C | | 132.0, C | | 125.8, C |
| 14-NH | 10.63, br. s | | | | 11.9, br. s | |
| 15-NH | 10.72, br. s | | 10.76, br. s | | 11.4, br. s | |
| 2-$OCH_3$ | 3.74, s | 55.3, $CH_3$ | 3.81, br. s | 55.4, $CH_3$ | 3.81, s | 55.3, $CH_3$ |
| 10-$OCH_3$ | 3.73, s | 55.3, $CH_3$ | 3.77, s | 55.2, $CH_3$ | 3.83, s | 55.3, $CH_3$ |
| 14-$CH_2$ | | | 5.41, s | 65.5, $CH_3$ | | |
| OH | | | 6.31, s | | | |

[a] $^1$H and $^{13}$C NMR were recorded at 400 and 100 MHz, respectively; [b] $^1$H and $^{13}$C NMR were recorded at 600 and 150 MHz, respectively.

structures –$CH_2CH_2N$– were indicated by the $^1$H–$^1$H COSY spectrum. These two partial structures were connected to the C-5a and C-8a, respectively, which were confirmed by the HMBC correlations of H-6 with C-5a and H-7 with C-8a. The HMBC correlations of H-6 with C-7 and H-7 with C-6 assigned the connection of C-6–N–C-7. The remaining sp$^3$ methine signal at ($\delta_H$ 3.63, 1H, br. s, and $\delta_C$ 56.8) was assigned as C-13 by the HMBC correlations of the proton signal at $\delta_H$ 3.63 with C-6, C-7, C-13a, and C-13b, along with H-6 and H-7 with this methine carbon ($\delta_C$ 56.8), implying that this remaining carbon (C-13) was connected among C-13a, C-13b, and N atom. Thus, the planar structure of **1** was assigned, and which was consistent with the molecular formula.

It was interested that this planar structure was a symmetrical structure. The value of [$\alpha$] was zero as well as two peaks were observed in the LC-CD spectrum (see supplementary material) suggested that this compound was a racemic mixture not an enantiomer. The chemical shifts of C-5 and C-8, C-13a and C-13b located at the symmetrical positions showed significant difference indicated that the structure of **1** should be not a symmetrical molecular in the stereochemical structure. Therefore, compound **1** was elucidated as shown in the figure and named trifiline A.

High resolution ESIMS of compound **2** gave the [M + H]$^+$ quasi-molecular ion peak at *m/z* 404.1972 consistent with a molecular formula of $C_{24}H_{25}N_3O_3$, which has 30 mass units greater than that of **1**. The $^1$H and $^{13}$C NMR data (Table 1) of **2** was closely similar to those of **1** except for the disappearance of NH-14 signal and the presence of a hydroxymethyl group signals [$\delta_H$ 5.41 (2H, s), 6.31 (the proton of OH group, 1H, s), and $\delta_C$ 65.5] in **2**. The HMBC correlations of $CH_2$-14 with C-1a and C-13a implied that the carbon of the hydroxymethyl group was connected to N-14. The value of [$\alpha$] in **2** was also zero suggested that **2** was racemic mixture. Therefore, the structure of **2** was elucidated as shown in the figure and named trifiline B.

Trifiline C (**3**) was isolated as yellow amorphous solid. Its molecular formula was established as $C_{23}H_{19}N_3O_3$ on the basis a quasi-molecular ion peak at *m/z* 408 [M + Na]$^+$ in the ESI mass spectrum in combination with the $^{13}$C NMR and DEPT spectra. Comparison of the $^1$H and $^{13}$C NMR data (Table 1) of **3** with those of **1** indicated that both structure were similar. The main differences between them were the absence of two sp$^3$ methylenes and a sp$^3$ methine (C-13) and the appearance of two sp$^2$ aromatic quaternary carbons and an aromatic methine ($\delta_H$ 7.32, 1H, br. s, and $\delta_C$ 93.7) in **3**. In addition, the carbon chemical shifts of C-8a and C-12a were down shifted ca. Δ16.5 and 3.8 ppm, respectively, while C-9a, C-13a, and C-13b were up shifted ca. Δ5.3, 6.3, and 5.5 ppm, respectively. The aforementioned facts suggested that the D ring of **3** were oxidized and rearranged to a $\alpha,\beta$-unsaturated amides ring. This was confirmed by the HMBC correlations of H-8 with C-7, C-9a, and C-13b, and H-6 to C-7 and C-13. Thus, this structure of **3** was determined and named trifiline C.

Trigonoine C (**7**) was isolated from *Trigonostemon lii*, yellow solid. The molecular weight was assigned as 394 on the basis of positive ESIMS at *m/z* 395.7 [M + H]$^+$ and positive FABMS *m/z* 395 [M + H]$^+$. Then its molecular formula was deduced as $C_{26}H_{38}N_2O$ by combination with the $^{13}$C NMR and DEPT spectra. IR absorptions at 3419, 2925, 2853, 1630, 1568, and 1453 cm$^{-1}$ indicated the presence of NH, $CH_2$, and aromatic functionalities, respectively. The $^1$H and $^{13}$C NMR data (Table 2) was observed the presence of a 7-methoxy $\beta$-carboline unit, which was further confirmed by the 2D NMR correlations (Figure 1). Apart from this moiety, a =CH–$(CH_2)_{12}$–$CH_3$ fatty chain was confirmed by the $^1$H–$^1$H COSY and HMBC correlations. This fatty chain was connected

**Table 2. NMR data for trigonoine C (7) in acetone-$d_6$ ($\delta$ in ppm, $J$ in Hz).**

| No. | $\delta_H$ [a] | $\delta_C$ [b] | No. | $\delta_H$ [a] | $\delta_C$ [b] |
|---|---|---|---|---|---|
| 1 | | 146.2 C | 3' | 3.10, m | 34.5 $CH_2$ |
| 3 | 8.22, d (5.3) | 139.1 CH | 4' | 1.86, m | 29.2 $CH_2$ |
| 4 | 7.77, d (5.3) | 112.5 CH | 5' | 1.23–1.42, overlap | 29.7 $CH_2$ |
| 4a | | 129.2 C | 6' | 1.23–1.42, overlap | 29.7 $CH_2$ |
| 5 | 8.03, d (8.6) | 123.1 CH | 7' | 1.23–1.42, overlap | 30.3 $CH_2$ |
| 5a | | 116.4 C | 8' | 1.23–1.42, overlap | 30.3 $CH_2$ |
| 6 | 6.85, dd (8.6, 2.1) | 110.1 CH | 9' | 1.23–1.42, overlap | 30.4 $CH_2$ |
| 7 | | 161.7 C | 10' | 1.23–1.42, overlap | 29.5 $CH_2$ |
| 8 | 7.06, d (2.1) | 96.5 CH | 11' | 1.86, m | 29.3 $CH_2$ |
| 8a | | 143.2 C | 12' | 1.23, m | 32.6 $CH_2$ |
| 9a | | 135.3 C | 13' | 1.25, m | 23.2 $CH_2$ |
| 1' | 5.32, m | 130.5 CH | 14' | 0.84, q (7.0) | 14.3 $CH_2$ |
| 2' | 1.99, m | 27.7 $CH_2$ | 7-OMe | 3.87, s | 55.8 $CH_3$ |

[a]Recorded at 400 MHz; [b]Recorded at 100 MHz to C-1 of $\beta$-carboline unit by the HMBC correlations of H-3, H-2', and H-3' with C-1. Thus, this structure of **7** was determined as shown in the figure and named trigonoine C.

Three known $\beta$-carboline alkaloids isolated from *T. filipes* were indentified as trigonostemine A (**4**),[2e] norharmine (**5**),[4] and harmine (**6**)[5] by comparison of their NMR data with those reported.

Trifilines A (**1**) and B (**2**) were tested for cytotoxicity against C8166 cells ($CC_{50}$), and anti-HIV-1 activity was evaluated by inhibition assay for the cytopathic effects of HIV-1 ($EC_{50}$), using AZT as a positive control. Both of them showed weak anti-HIV-1 activities with the $EC_{50}$ value of 54.61 $\mu$g/mL and 9.75 $\mu$g/mL, along with TI (Therapeutic index) values of 1.52 and 1.42, respectively.

**Figure 1.** Key 2D NMR correlations of compounds **1**, **3**, and **7**

## Experimental Section

**General Experimental Procedures.** Optical rotations were obtained on a JASCO DIP-370 digital polarimeter. IR spectra were measured in a Bio-Rad FTS-135 spectrometer with KBr pellets, and UV data were measured using a UV-210A spectrometer. CD spectra were recorded with an Applied Photophysics Chirascan spectrometer. 1D and 2D NMR spectra were measured on Bruker AM-400, DRX-500, and AV-600 NMR spectrometers using TMS as an internal standard. ESIMS were recorded using a Finnigan MAT 90 instrument and a VG Auto Spec-3000 spectrometer. Column chromatography was performed on Si gel H (10–40 $\mu$m; Qingdao Marine Chemical Factory) and Sephadex LH-20 (40–70 $\mu$m, Amersham Pharmacia Biotech AB, Uppsala, Sweden). MPLC was performed on Büchi Sepacore System (Büchi Labortechnik AG, Switzerland), and columns packed with Chromatorex $C_{18}$ (40–75 $\mu$m, Fuji Silysia Chemical Ltd., Japan). Preparative HPLC was performed by using an Agilent 1200 series system equipped with a Zorbax XDB-C18, 9.4 mm × 150 mm column.

**Plant Material.** The plant of *T. filipes* were collected in Nonggang, Guangxi Province, China, in November, 2009, and the plant sample was identified by Mr. Mao-Xin Lu of Nonggang National Nature Reserve. The leaves of *T. lii* were collected in Xishuangbanna, Yunnan Province, China, in November, 2008, and the plant sample was identified by Prof. Shun-Cheng Zhang of Xishuangbanna Institute of Botany, Chinese Academy of Sciences (CAS). Both of the voucher specimens (KIB 09110206 and KIB 08110211) were deposited at the State Key Laboratory of Phyotochemistry and Plant Resources in West China, Kunming Institute of Botany, Chinese Academy of Science (CAS).

**Extraction and Isolation.** The air-dried powder (8.0 kg) of *T. filipes* was extracted three times with MeOH at room temperature to give a crude extract, and then partitioned between EtOAc and $H_2O$. The EtOAc-soluble residue (200 g) was subjected to CC on silica gel using petroleum ether/acetone (10:1 to 1:2) to yield 10 fractions (Fr.1–Fr.10). Repeated column chromatography of Fr.3 over silica gel (petroleum ether–EtOAc, 1:1; $CHCl_3$–MeOH, 30:1) gave **3** (3 mg), **4** (5 mg), **5** (5 mg), and **6** (4 mg), respectively. Fr.4 was separated using reversed-phase MPLC by a gradient of $H_2O$–MeOH and Sephadex LH-20 ($CHCl_3$–MeOH), to afford **1** (100 mg) and **2** (15 mg). The EtOAc-soluble fraction of the MeOH extract of the leaves of *T. lii* was subjected to column chromatography on silica gel, RP-18, and Sephadex LH-20, as well as semi-preparative HPLC to afford **7** (4 mg).

**Trifiline A (1):** yellow solid; $[\alpha]_D^{22}$ 0 (*c* 0.28, MeOH); UV (DMSO) $\lambda_{max}$ (log $\varepsilon$) 198 (3.21), 209 (3.26), 246 (3.37), 271 (3.52), and 301 (3.60) nm; IR (KBr) $\nu_{max}$ 3349, 2899, 2840, 1631, 1499, 1475, 1440, 1366, 1331, 1295, 1272, 1234, 1198, 1150, 1112, 1027, 1013, 825, and 817 $cm^{-1}$; $^1H$ and $^{13}C$ NMR data, see Table 1; positive ESIMS *m/z* 374.2 $[M + H]^+$; positive HRESIMS *m/z* 374.1875 $[M + H]^+$ (calcd for $C_{23}H_{24}N_3O_2$, 374.1868).

**Trifiline B (2):** yellow solid; $[\alpha]_D^{22}$ 0 (*c* 0.34, MeOH); UV (DMSO) $\lambda_{max}$ (log $\varepsilon$) 228 (4.77), 267 (4.05), 297 (4.03), and 333 (3.50) nm; IR (KBr) $\nu_{max}$ 3422, 2904, 2834, 1630, 1492,

1472, 1439, 1367, 1310, 1274, 1230, 1201, 1156, 1031, 1013, and 805 $cm^{-1}$; $^{1}H$ and $^{13}C$ NMR data, see Table 1; positive ESIMS *m/z* 404 $[M + H]^+$, 426 $[M + Na]^+$, 807 $[2M + H]^+$, 829 $[2M + Na]^+$; positive HRESIMS *m/z* 404.1972 $[M + H]^+$ (calcd for $C_{24}H_{26}N_3O_3$, 404.1974).

**Trigonoine C (7):** yellow solid; UV (DMSO) $\lambda_{max}$ (log $\varepsilon$) 196 (4.23), 209 (4.16), 241 (4.48), and 301 (4.11) nm; IR (KBr) $\nu_{max}$ 3419, 2925, 2853, 1630, 1568, 1453, 1426, 1383, 1325, 1277, 1235, 1200, 1162, 1032, 822, and 803 $cm^{-1}$; $^{1}H$ and $^{13}C$ NMR data, see Table 2; positive ESIMS *m/z* 395.7 $[M + H]^+$, positive FABMS *m/z* 395 $[M + H]^+$.

**Anti-HIV-1 Assay.** Cytotoxicity against C8166 cells ($CC_{50}$) was assessed using the MTT method, and anti-HIV-1 acitivity was evaluated by the inhibition assay for the cytopathic effects of HIV-1 ($EC_{50}$).[6]

## Electronic Supplementary Material

Supplementary material is available in the online version of this article at http://dx.doi.org/ 10.1007/s13659-012-0028-x and is accessible for authorized users.

## Acknowledgments

This work was supported financially by the National Natural Science Foundation of China (30830114 and 21072199), the Ministry of Science and Technology of China (2009CB522300 and 2009CB940900), the Natural Science Fund of Yunnan Province (2009CD112), the Foundation of Chinese Academy of Sciences to H.P. He, the Yong Academic and Technical Leader Raising Foundation of Yunnan Province (2010CI047).

**Open Access** This article is distributed under the terms of the Creative Commons Attribution License which permits any use, distribution, and reproduction in any medium, provided the original author(s) and source are credited.

## Electronic supplementary material

Supplementary material, approximately 1.91 MB.
