# Supplementary material for: β-carboline alkaloids from Trigonostemon filipes and Trigonostemon lii
Source: Nat Prod Bioprospect. 2012 May 3;2(3):126–9. doi: 10.1007/s13659-012-0028-x (PMC4131596; doi:10.1007/s13659-012-0028-x)

Electronic Supplementary Material

β-Carboline alkaloids from *Trigonostemon filipes* and *Trigonostemon lii*

Shi-Fei LI,[a,b] Yuan-Yuan CHENG,[a,b] Yu ZHANG,[a] Shun-Lin LI,[a] Hong-Ping HE,[a,*] and Xiao-Jiang HAO[a,*]

[a]State Key Laboratory of Phytochemistry and Plant Resources in West China, Kunming Institute of Botany, Chinese Academy of Sciences, Kunming 650201, China

[b]Graduate University of Chinese Academy of Sciences, Beijing 100049, China

Received 28 March 2012; Accepted 22 April 2012

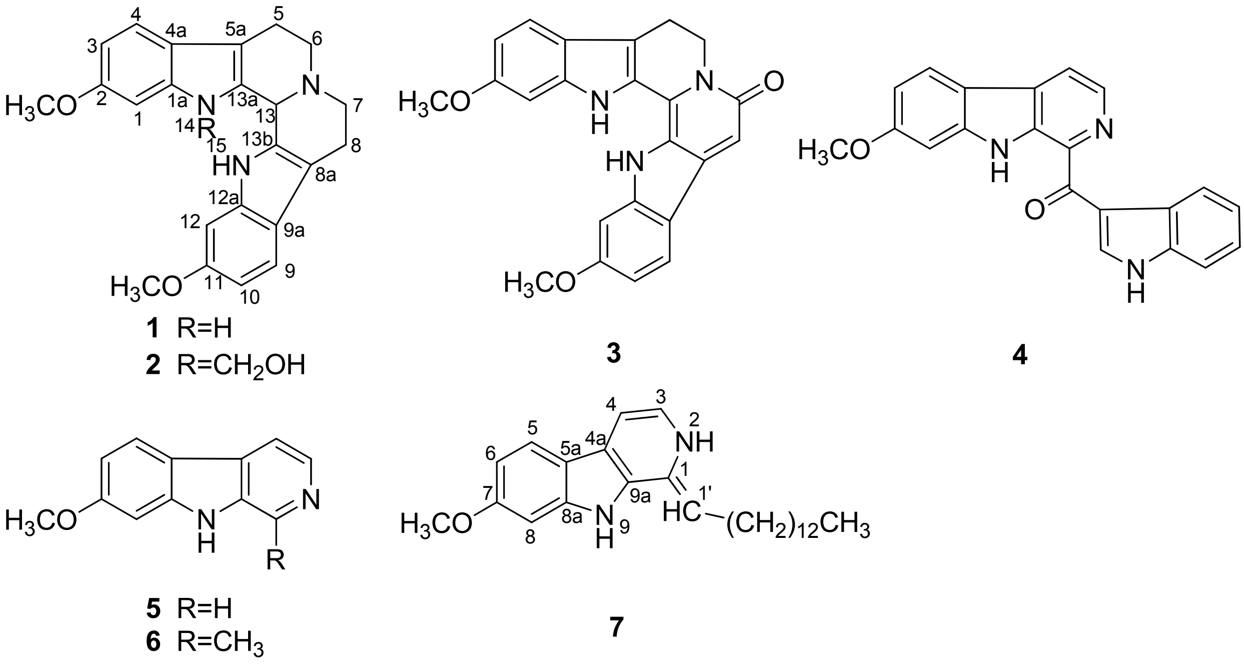

Structures of compounds **1**–**7**

*To whom correspondence should be addressed. E-mail: haoxj@mail.kib.ac.cn (X.J. Hao); hehongping@mail.kib.ac.cn (H.P. He).

Contents

S1. ESI-MS of trifiline A (**1**).

S2. HR-ESI-MS of trifiline A (**1**).

S3. IR spectrum of trifiline A (**1**).

S4. UV spectrum of trifiline A (**1**).

S5. ^{1}H NMR spectrum of trifiline A (**1**).

S6. ^{13}C NMR spectrum of trifiline A (**1**).

S7. HSQC spectrum of trifiline A (**1**).

S8. ^{1}H-^{1}H COSY spectrum of trifiline A (**1**).

S9. HMBC spectrum of trifiline A (**1**).

S10. ESI-MS of trifiline B (**2**).

S11. HR-ESI-MS of trifiline B (**2**).

S12. UV spectrum of trifiline B (**2**).

S13. IR spectrum of trifiline B (**2**).

S14. ^{1}H NMR spectrum of trifiline B (**2**).

S15. ^{13}C NMR spectrum of trifiline B (**2**).

S16. HSQC spectrum of trifiline B (**2**).

S17. ^{1}H-^{1}H COSY spectrum of trifiline B (**2**).

S18. HMBC spectrum of trifiline B (**2**).

S19. ROESY spectrum of trifiline B (**2**).

S20. ESI-MS of trifiline C (**3**).

S21. ^{1}H NMR spectrum of trifiline C (**3**).

S22. ^{13}C NMR spectrum of trifiline C (**3**).

S23. HSQC spectrum of trifiline C (**3**).

S24. HMBC spectrum of trifiline C (**3**).

S25. ESI-MS of trigonoine C (**7**).

S26. FAB-MS of trigonoine C (**7**).

S27. UV spectrum of trigonoine C (**7**).

S28. IR spectrum of trigonoine C (**7**).

S29. ^{1}H NMR spectrum of trigonoine C (**7**).

S30. ^{13}C NMR spectrum of trigonoine C (**7**).

S31. HSQC spectrum of trigonoine C (**7**).

S32. ^{1}H-^{1}H COSY spectrum of trigonoine C (**7**).

S33. HMBC spectrum of trigonoine C (**7**).

S34. LC-CD data analysis of trifiline A (**1**).

S1. ESI-MS of trifiline A (**1**).

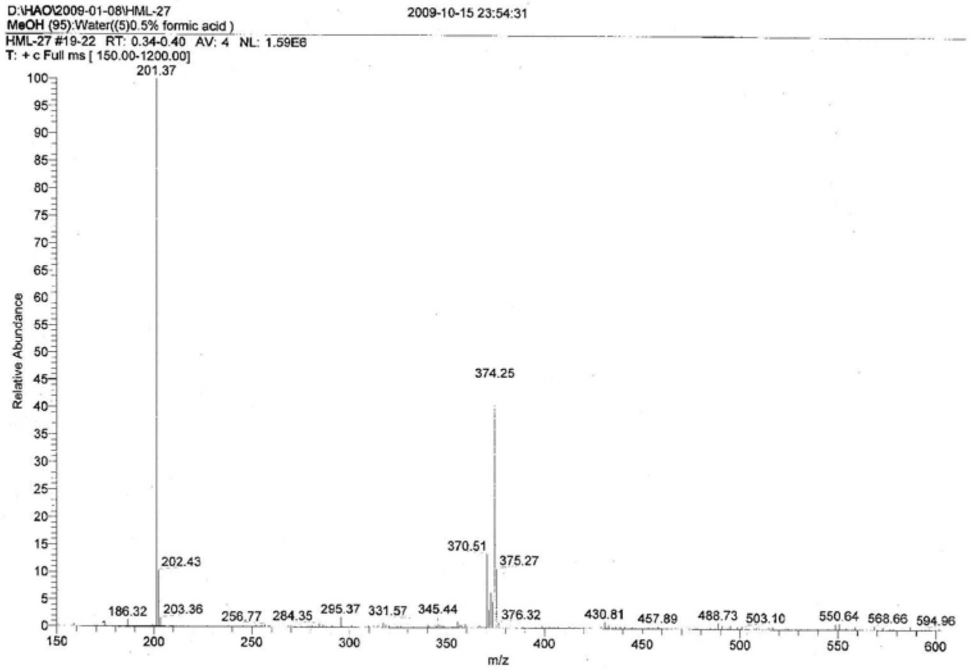

S2. HR-ESI-MS of trifiline A (**1**).

Acq. Date: Friday, February 10, 2012

Acq. Time: 13:27

Sample Name: 120210ESIA

Elemental composition calculator

Target m/z: +374.1875 amu

Tolerance: +10.0000 ppm

Result type: Elemental

Max num of results: 1000

Min DBE: -10.0000 Max DBE: +60.0000

Electron state: OddAndEven

Num of charges: 0

Add water: N/A

Add proton: N/A

File Name: 120210ESIA

	Elements	Min Number	Max Number
1	2H	0	0
2	Br	0	0
3	C	0	200
4	Cl	0	0
5	F	0	0
6	H	0	400
7	I	0	0
8	K	0	0
9	N	3	3
10	Na	0	0

Acq. Date: Friday, February 10, 2012 Acq. Time: 13:27

Sample Name: 120210ESIA

	Elements	Min Number	Max Number
11	O	0	3
12	P	0	0
13	Pt	0	0
14	S	0	0
15	Si	0	0

	Formula	Calculated m/z (amu)	mDa Error	PPM Error	DBE
1	C23 H24 N3 O2	374.1868	0.6477	1.7310	13.5

S3. IR spectrum of trifiline A (**1**).

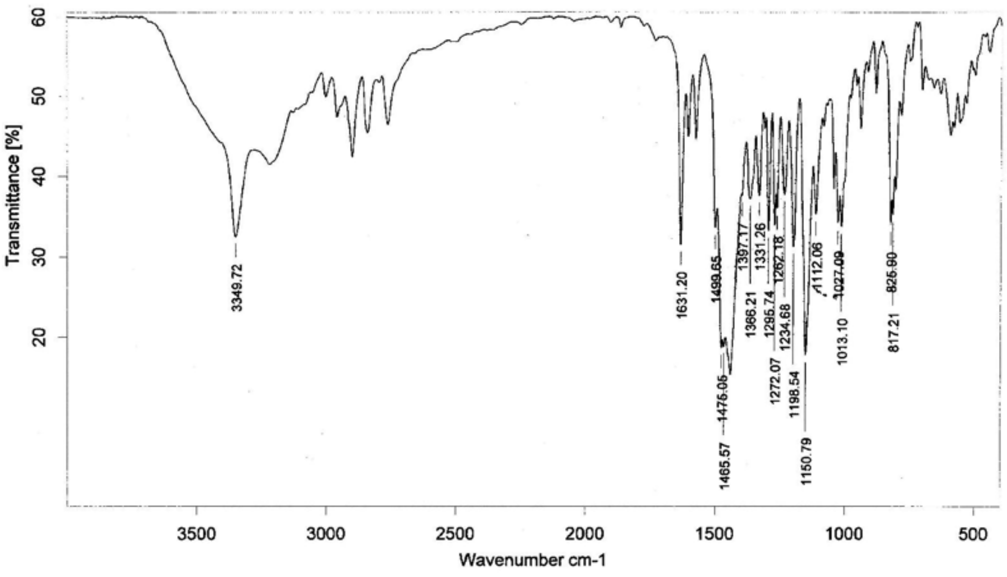

Sample : HST-1	Frequency Range : 399.246 - 3996.32	Measured on : 29/02/2012	
Technique : KBr压片	Resolution : 4	Instrument : Tensor27	Sample Scans : 16
Customer : 120229IR1	Zerofilling : 2	Acquisition : Double Sided,For	

S4. UV spectrum of trifiline A (**1**).

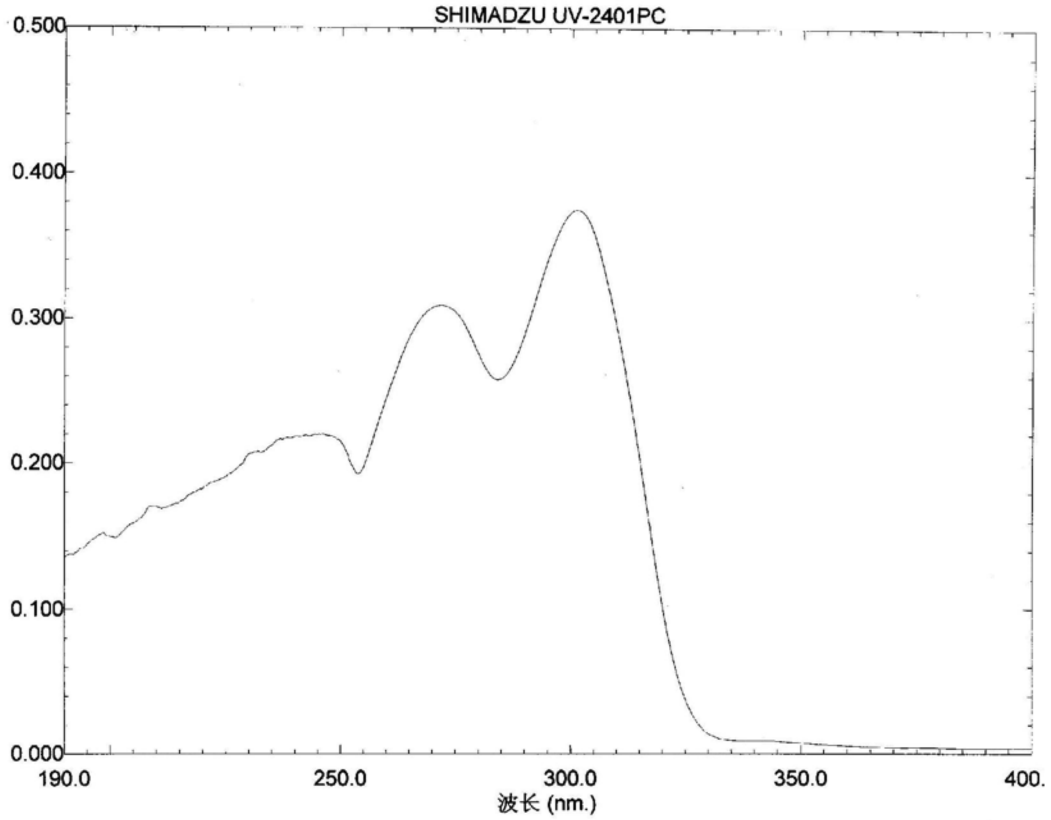

文件名:　12030100　　　　　12030100
样品名称：HST-1

样品浓度：0.0347毫克/毫升
溶剂：DMSO

创建于:　12:57　12-03-01
数据:　原始

测量模式:　Abs.
扫描速度:　中速
狭缝:　5.0
采样间隔:　0.2

否.	波长 (nm.)	Abs.
1	301.20	0.3751
2	271.80	0.3092
3	246.00	0.2201
4	209.20	0.1707
5	198.60	0.1523

S5. ^{1}H NMR spectrum of trifiline A (**1**).

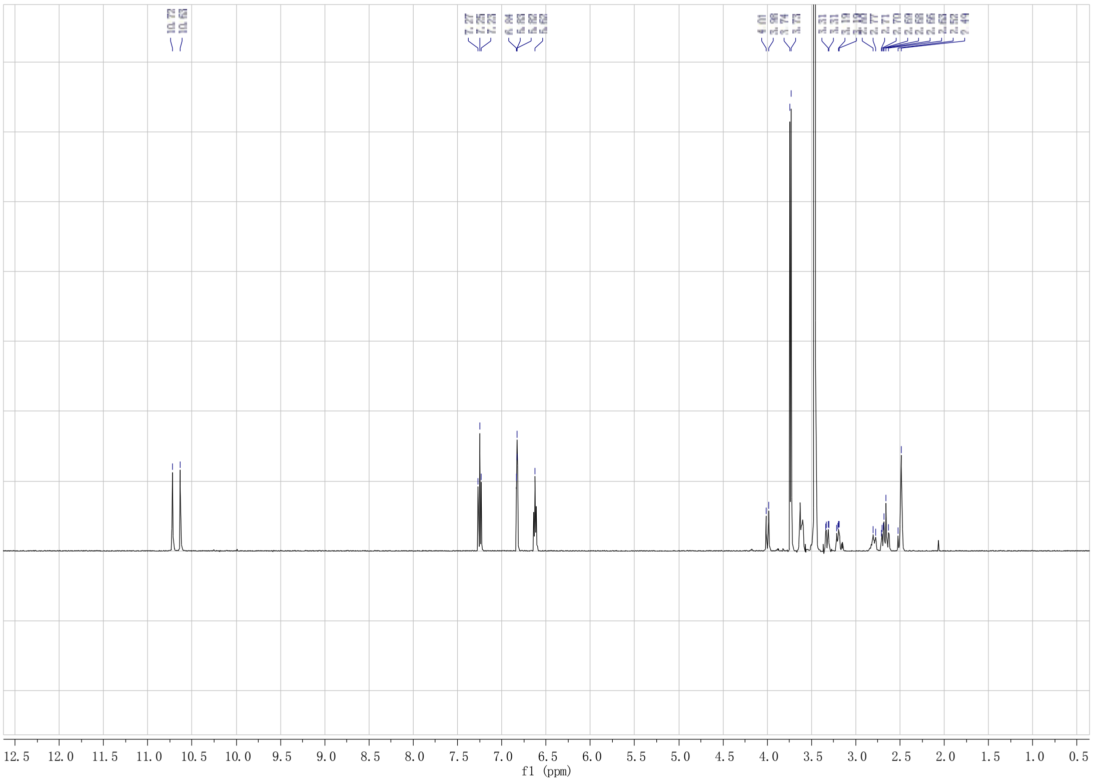

S6. ^{13}C NMR spectrum of trifiline A (**1**).

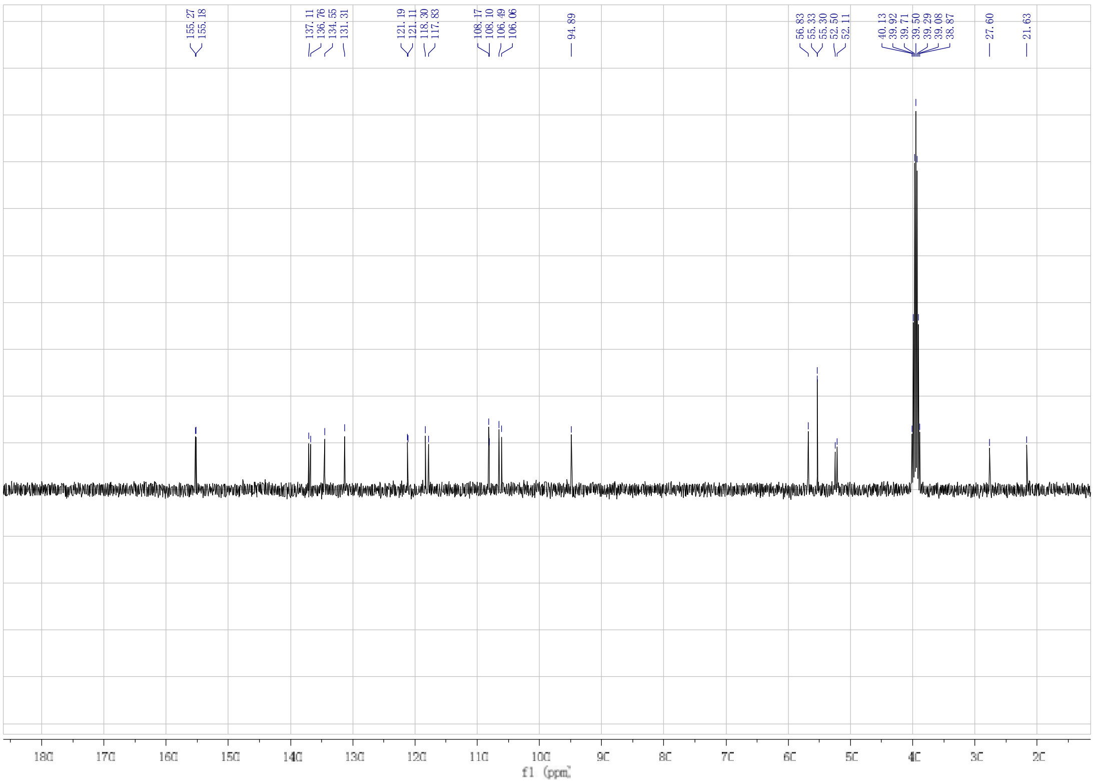

S7. HSQC spectrum of trifiline A (**1**).

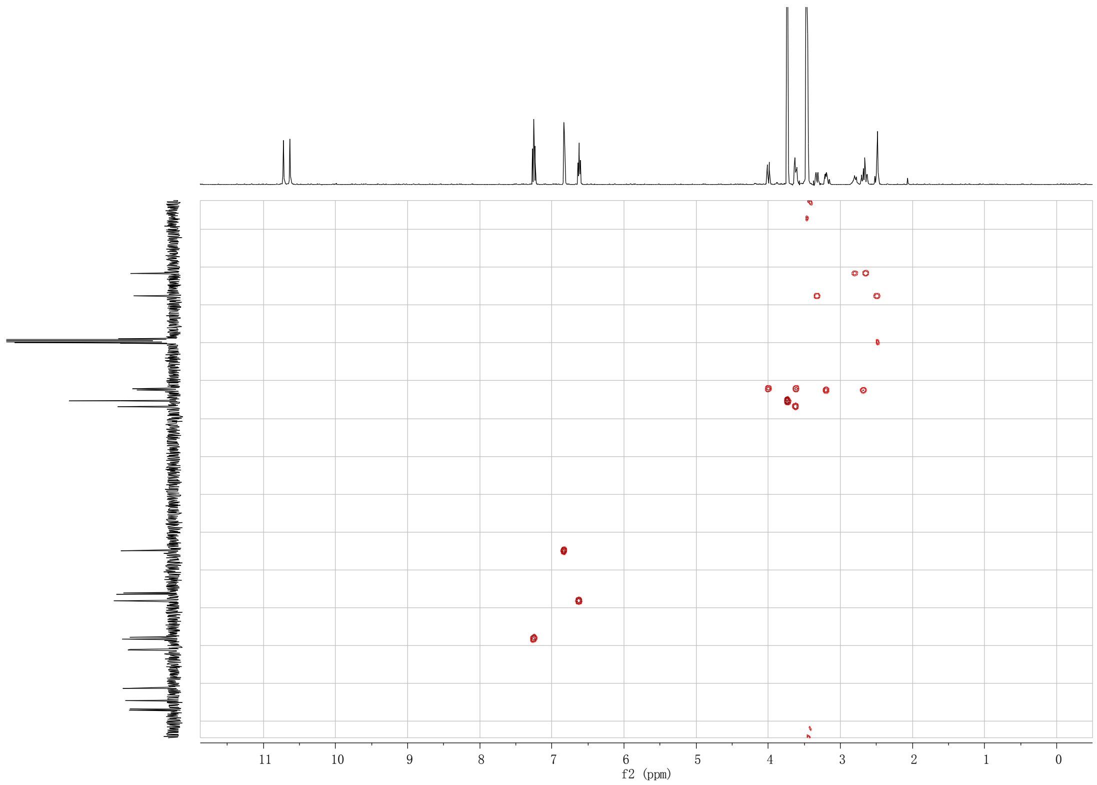

S8. ^{1}H-^{1}H COSY spectrum of trifiline A (**1**).

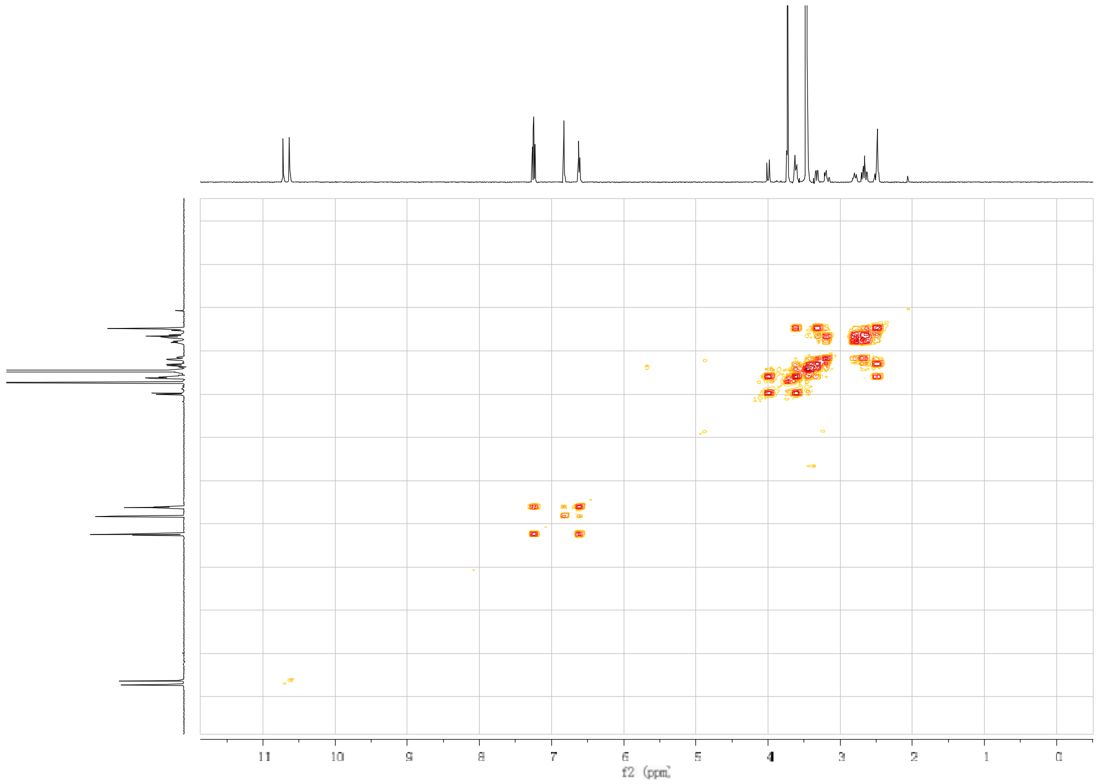

S9. HMBC spectrum of trifiline A (**1**).

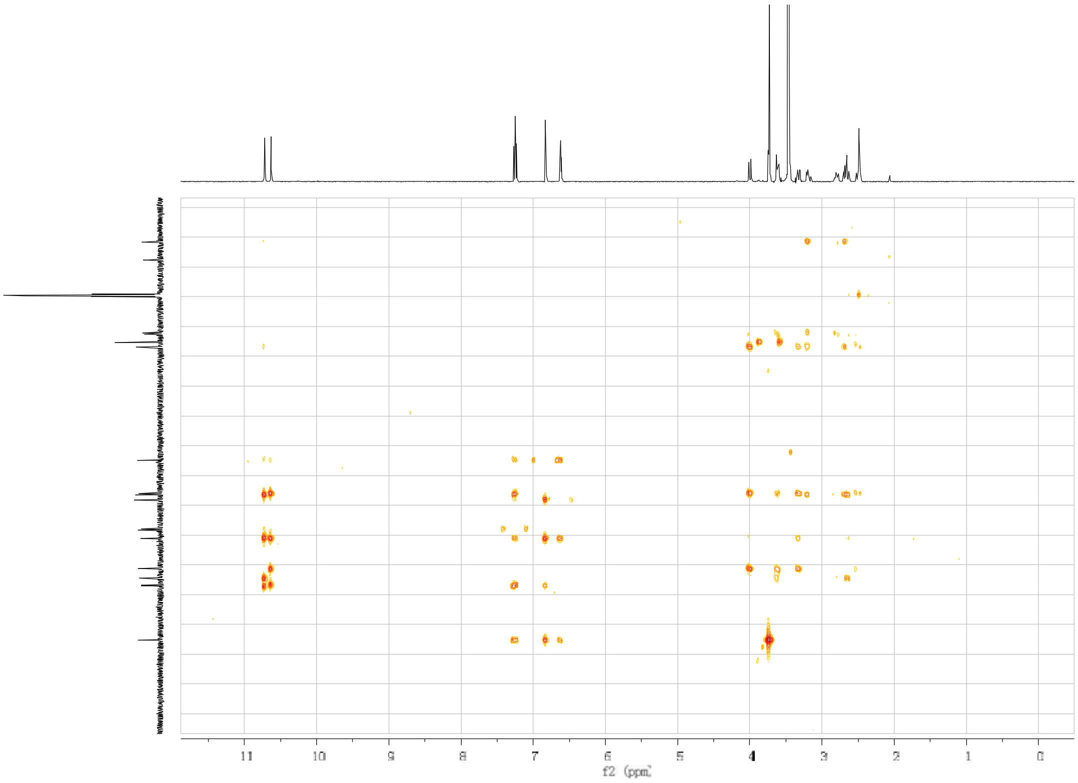

S10. ESI-MS of trifiline B (**2**).

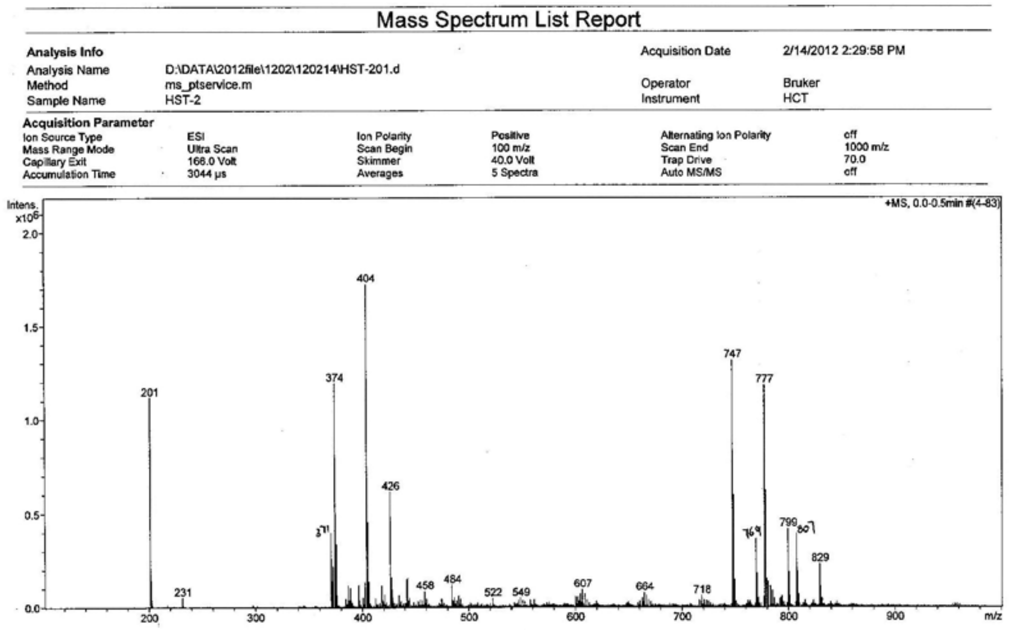

S11. HR-ESI-MS of trifiline B (**2**).

Date: Tuesday, February 14, 2012 Acq. Time: 15:11

Sample Name: 120215ESIA

Elemental composition calculator

Target m/z: +404.1972 amu
Tolerance: +10.0000 ppm
Result type: Elemental
Max num of results: 1000
Min DBE: -10.0000 Max DBE: +60.0000
Electron state: OddAndEven
Num of charges: 0
Add water: N/A
Add proton: N/A
File Name: 120215ESIA

	Elements	Min Number	Max Number
1	2H	0	0
2	Br	0	0
3	C	0	200
4	Cl	0	0
5	F	0	0
6	H	0	400
7	I	0	0
8	K	0	0
9	N	3	3
10	Na	0	0

Date: Tuesday, February 14, 2012 Acq. Time: 15:11

Sample Name: 120215ESIA

	Elements	Min Number	Max Number
11	O	1	3
12	P	0	0
13	Pt	0	0
14	S	0	0
15	Si	0	0

	Formula	Calculated m/z (amu)	mDa Error	PPM Error	DBE
1	C24 H26 N3 O3	404.1974	-0.2169	-0.5368	13.5

S12. UV spectrum of trifiline B (**2**).

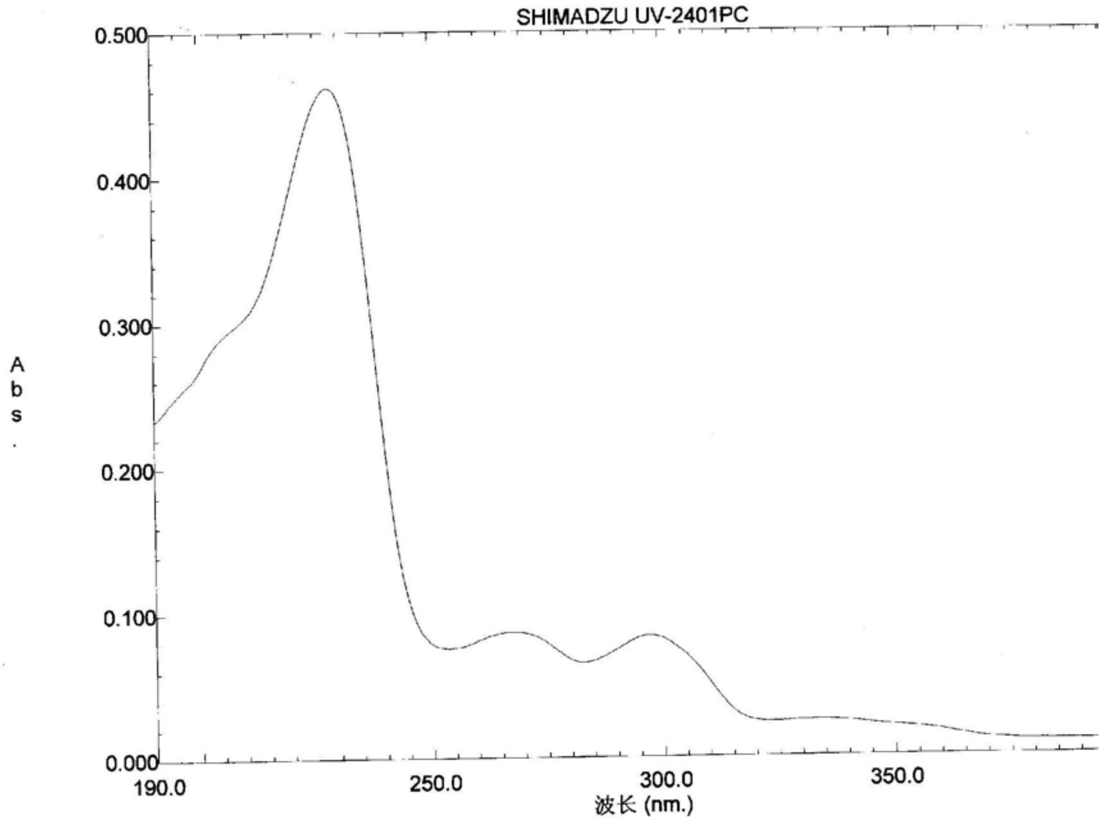

文件名: HST-2

HST-2 ———

样品浓度：0.0031毫克/毫升
溶剂：甲醇

创建于: 15:34 12-02-29
数据: 原始

测量模式: Abs.
扫描速度: 中速
狭缝: 5.0
采样间隔: 0.5

否.	波长 (nm.)	Abs.
1	333.00	0.0246
2	297.00	0.0835
3	267.50	0.0863
4	228.00	0.4615

S13. IR spectrum of trifiline B (**2**).

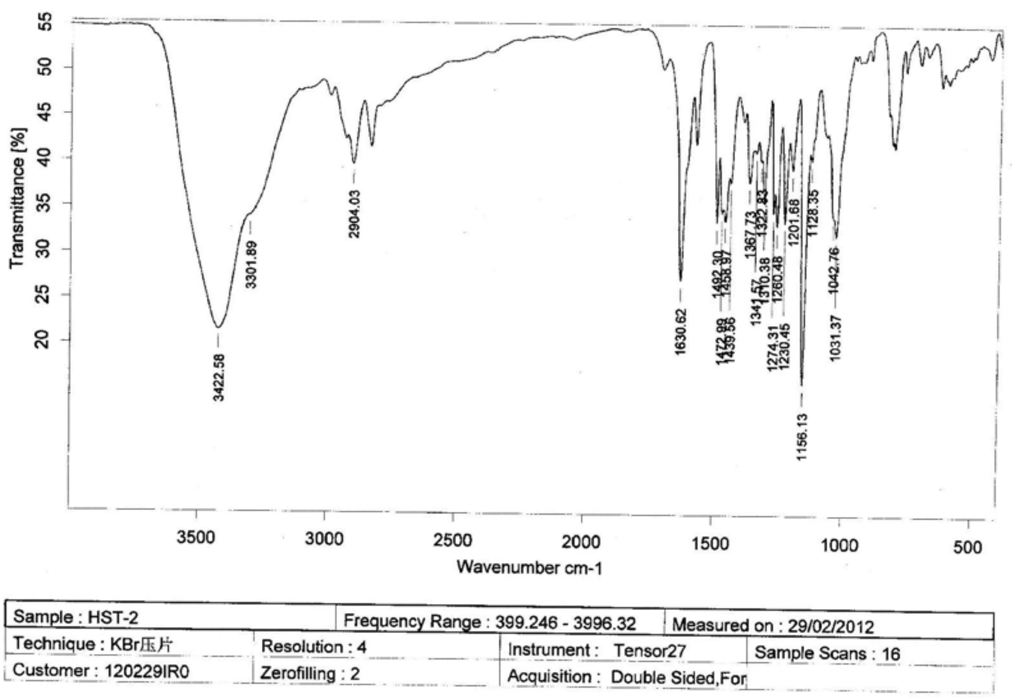

S14. ^{1}H NMR spectrum of trifiline B (**2**).

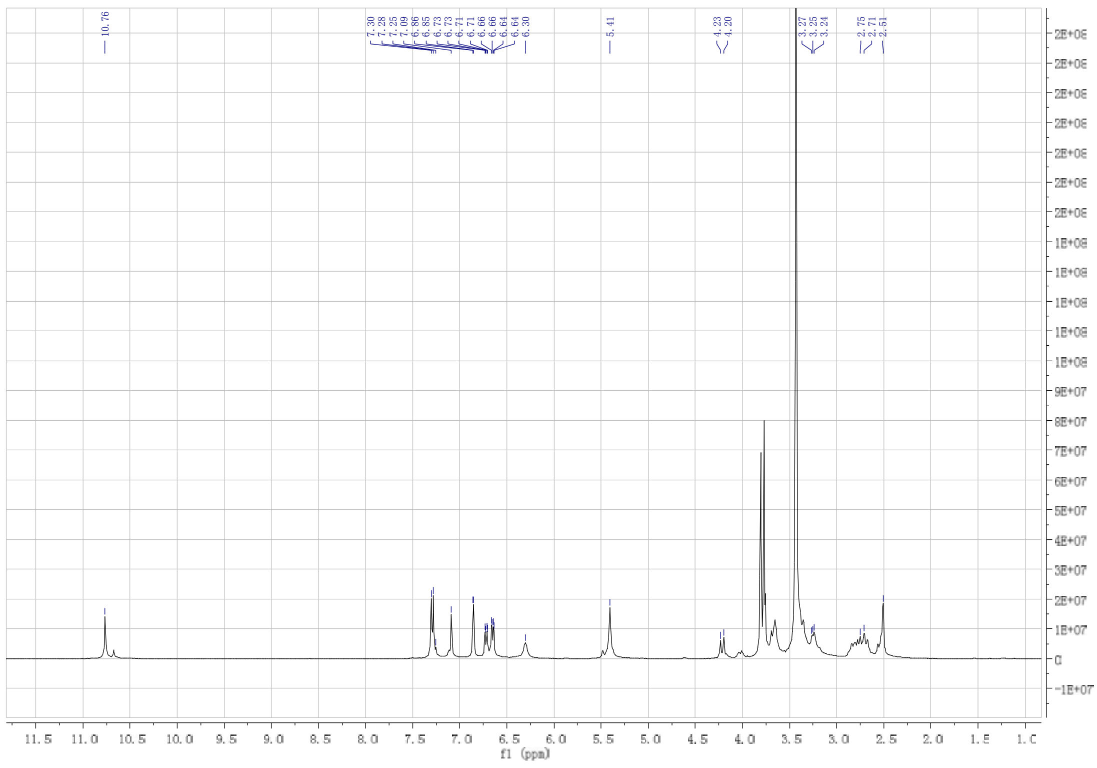

S15. ^{13}C NMR spectrum of trifiline B (**2**).

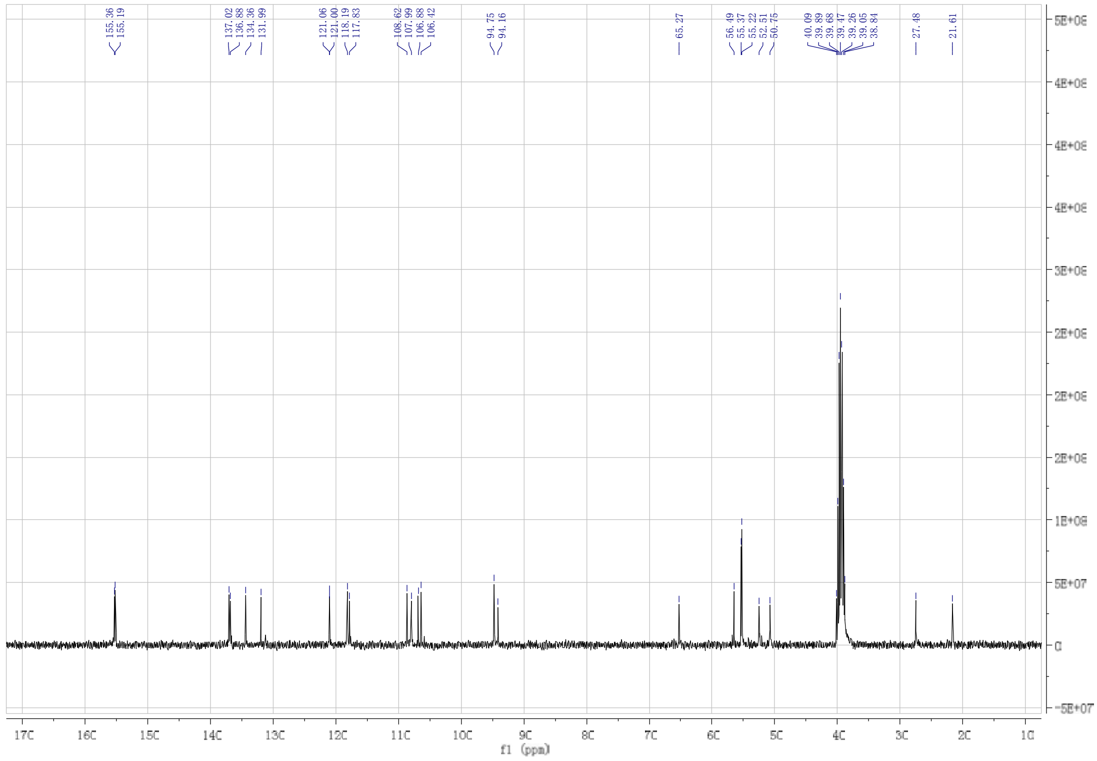

S16. HSQC spectrum of trifiline B (**2**).

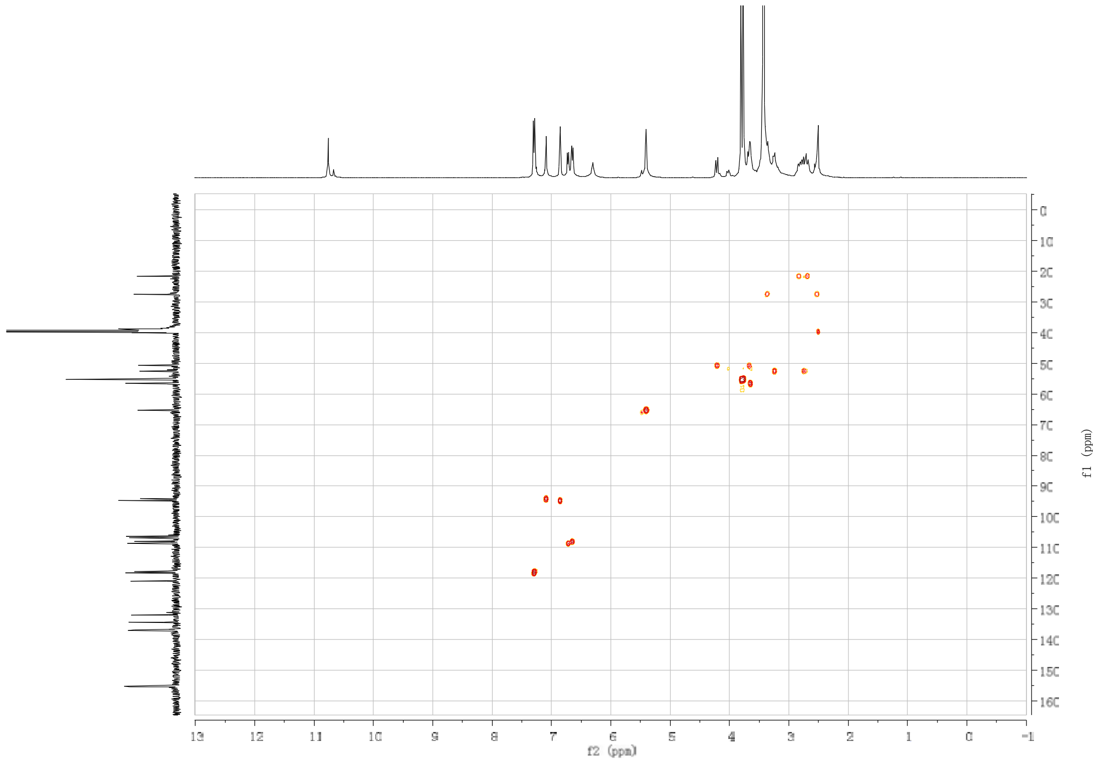

S17. ^{1}H-^{1}H COSY spectrum of trifiline B (**2**).

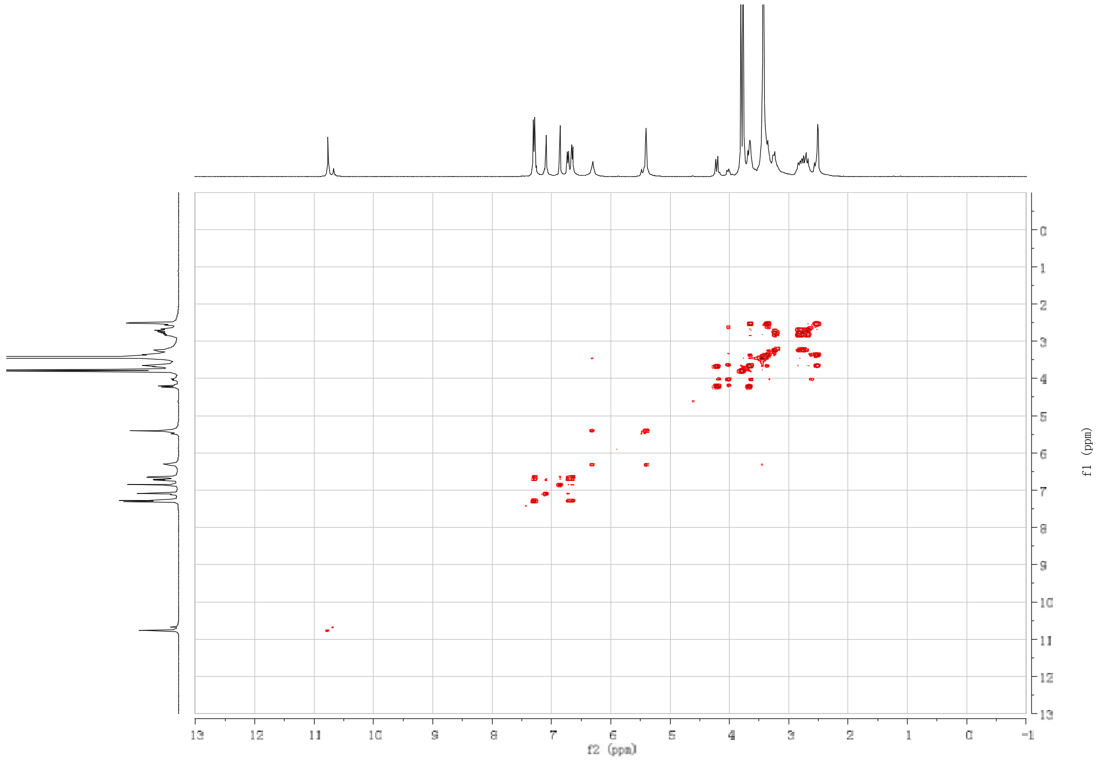

S18. HMBC spectrum of trifiline B (**2**).

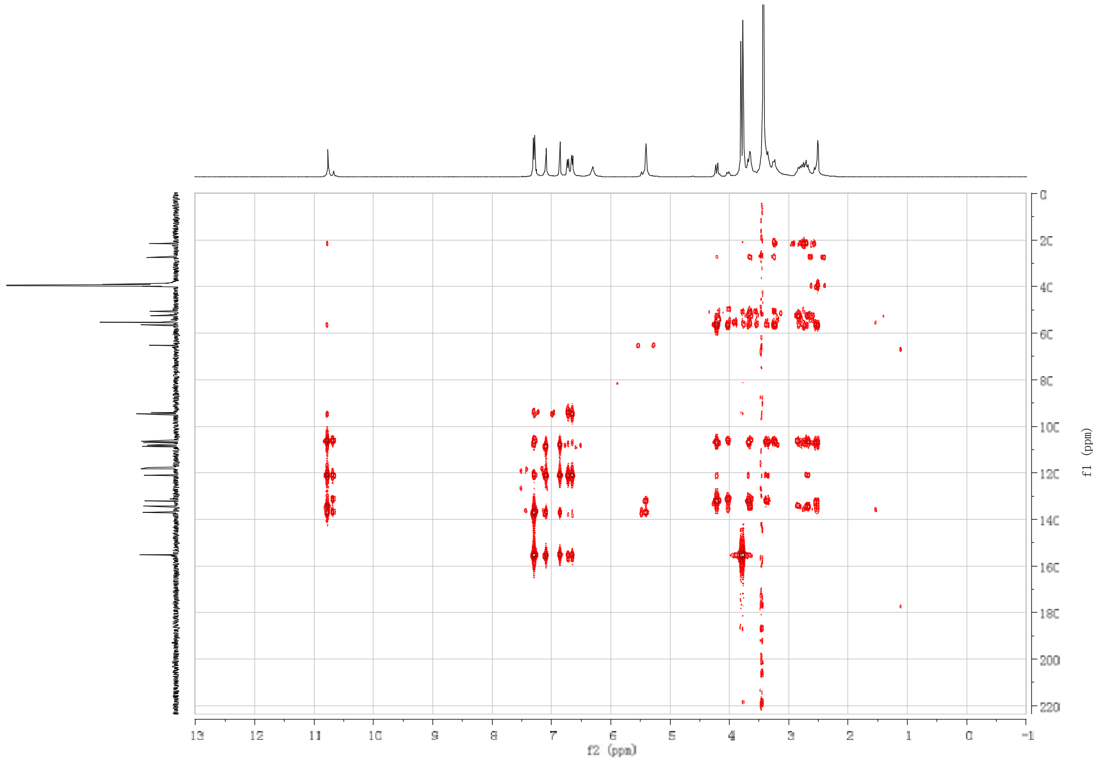

S19. ROESY spectrum of trifiline B (**2**).

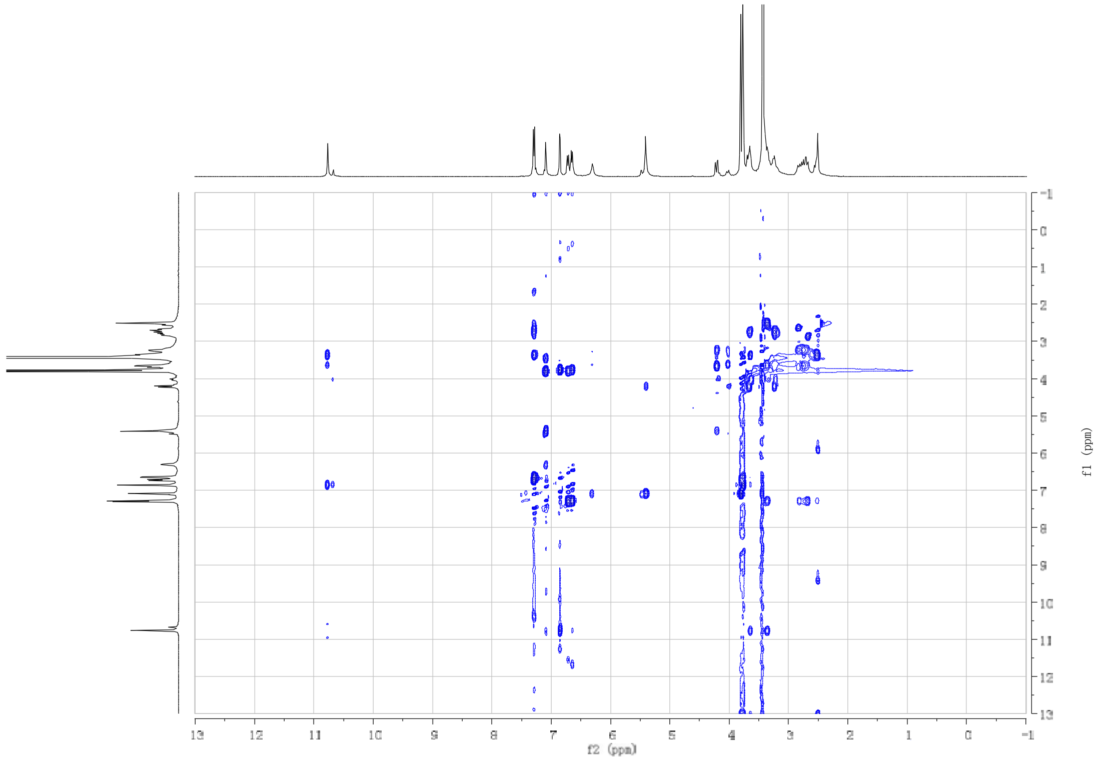

S20. ESI-MS of trifiline C (**3**).

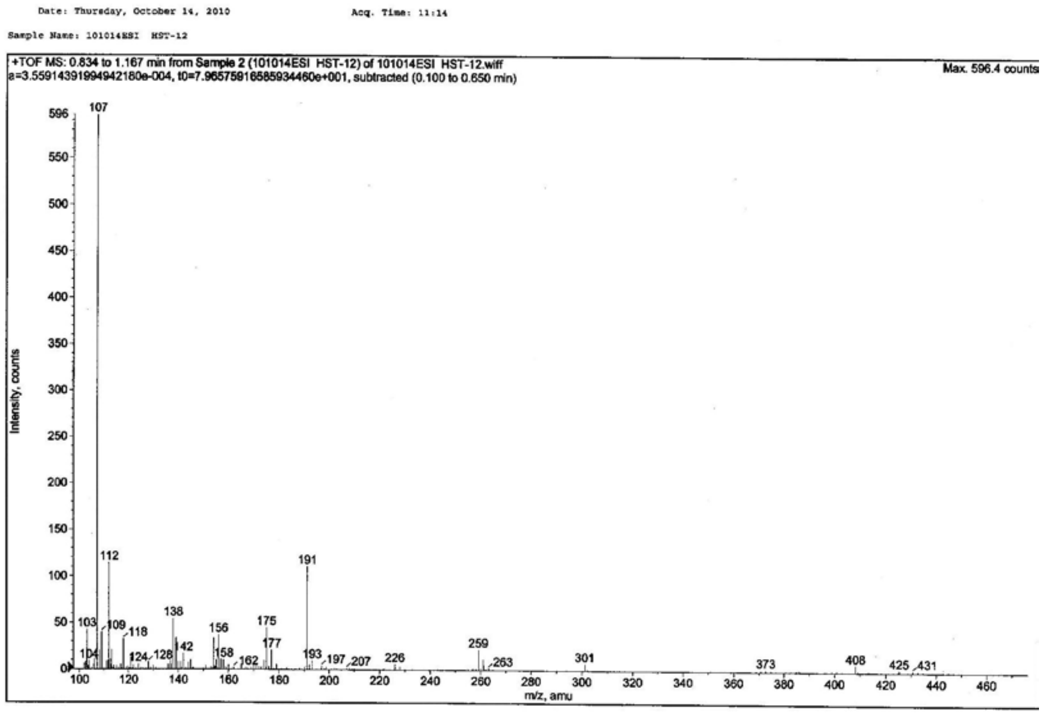

S21. ^{1}H NMR spectrum of trifiline C (**3**).

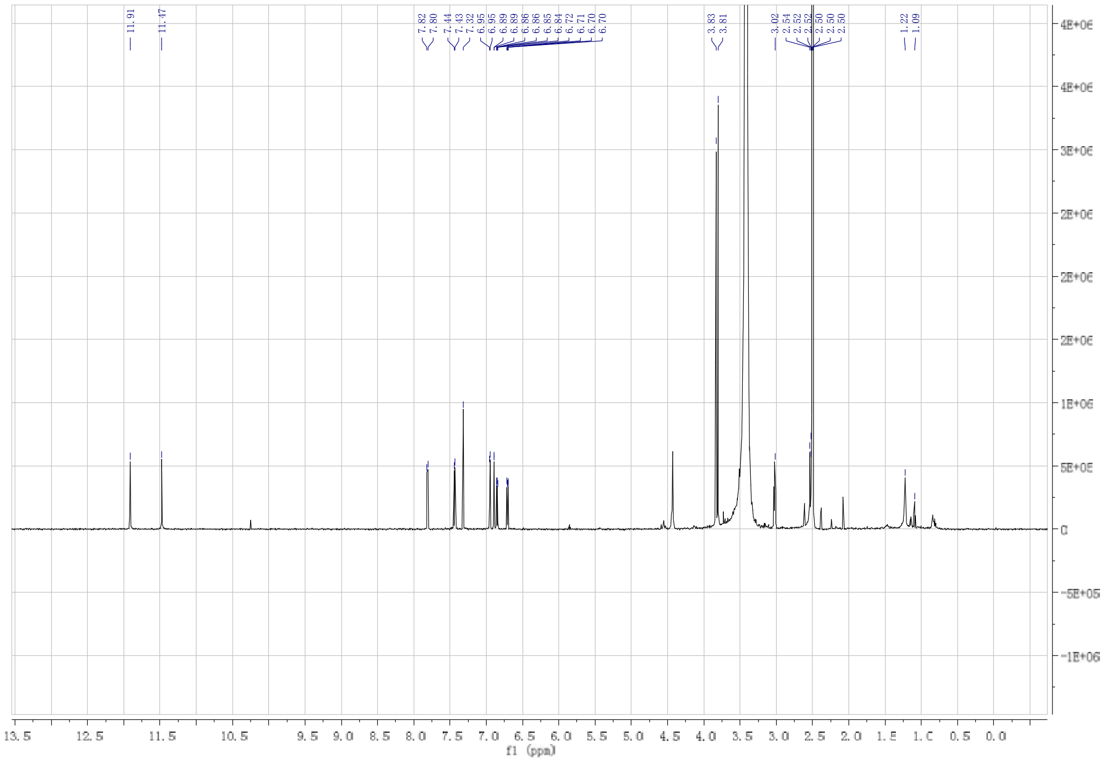

S22. ^{13}C NMR spectrum of trifiline C (**3**).

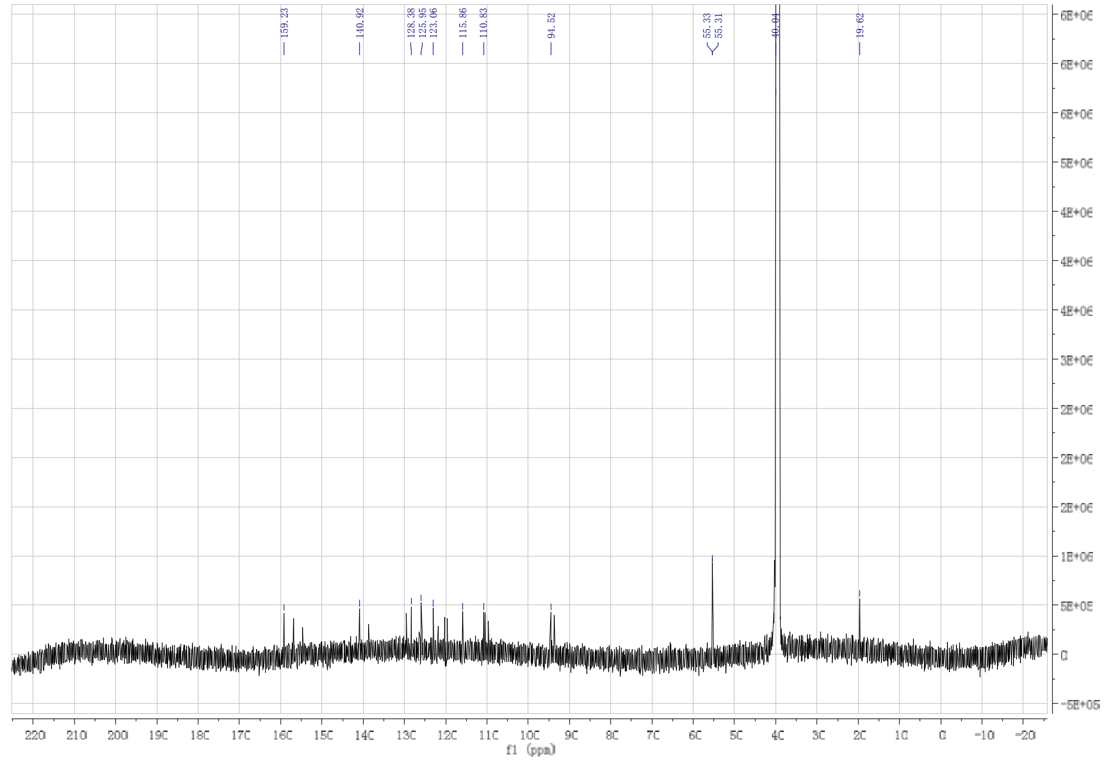

S23. HSQC spectrum of trifiline C (**3**).

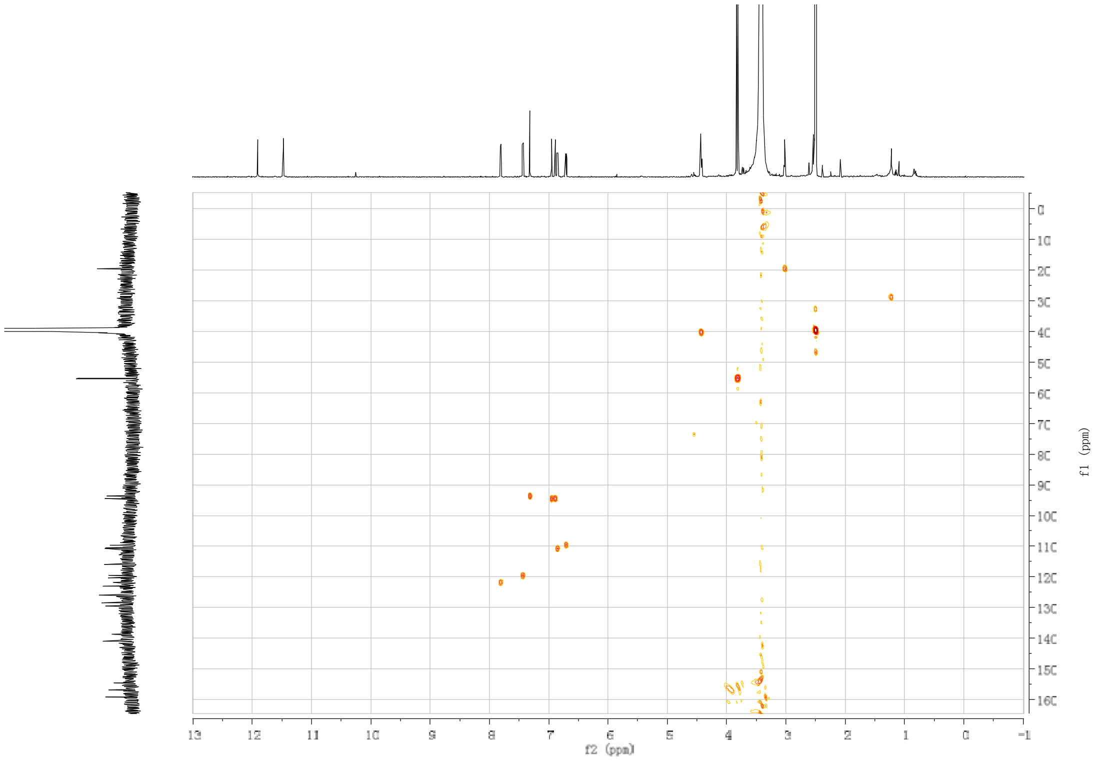

S24. HMBC spectrum of trifiline C (**3**).

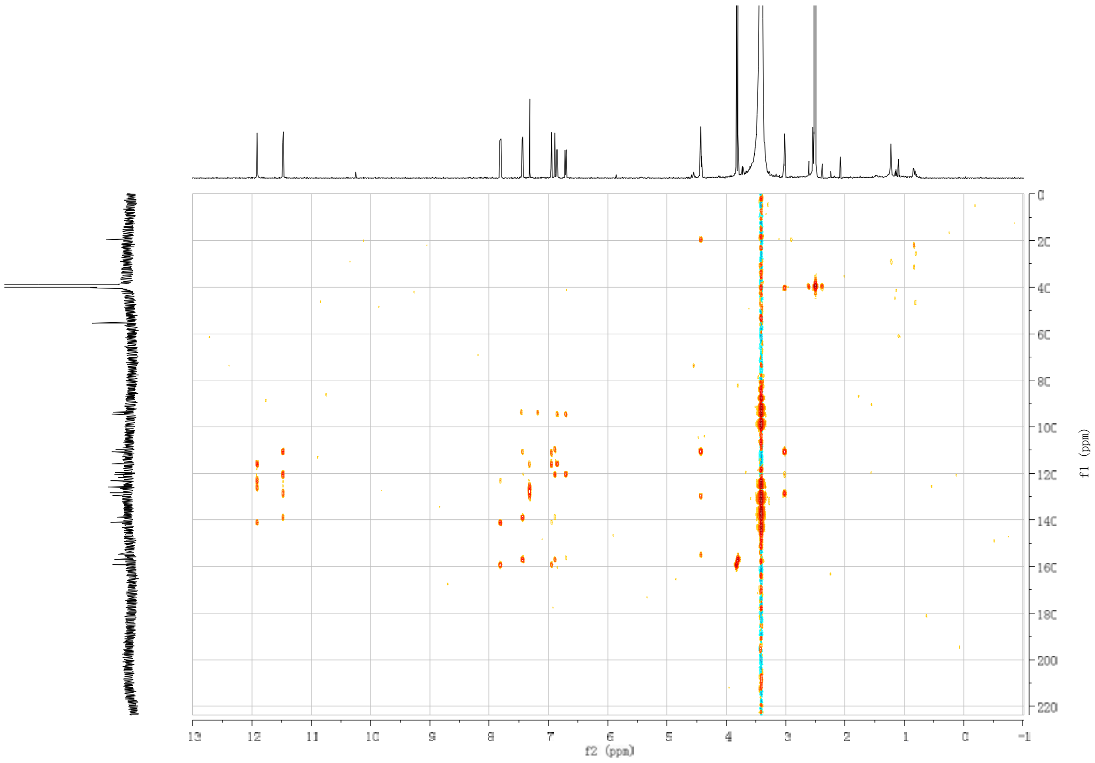

S25. ESI-MS of trigonoine C (**7**).

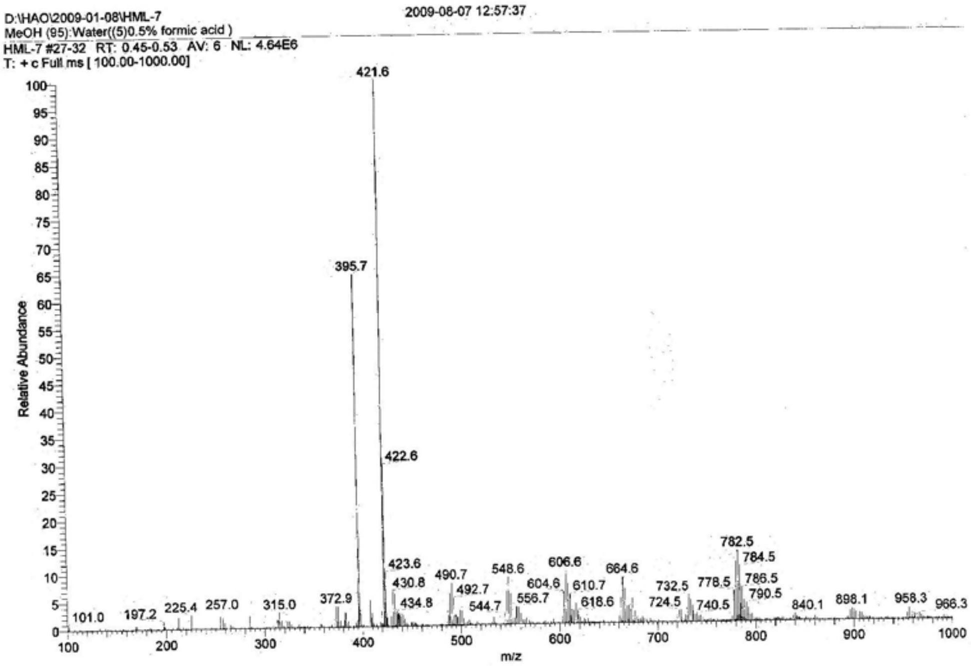

S26. FAB-MS of trigonoine C (**7**).

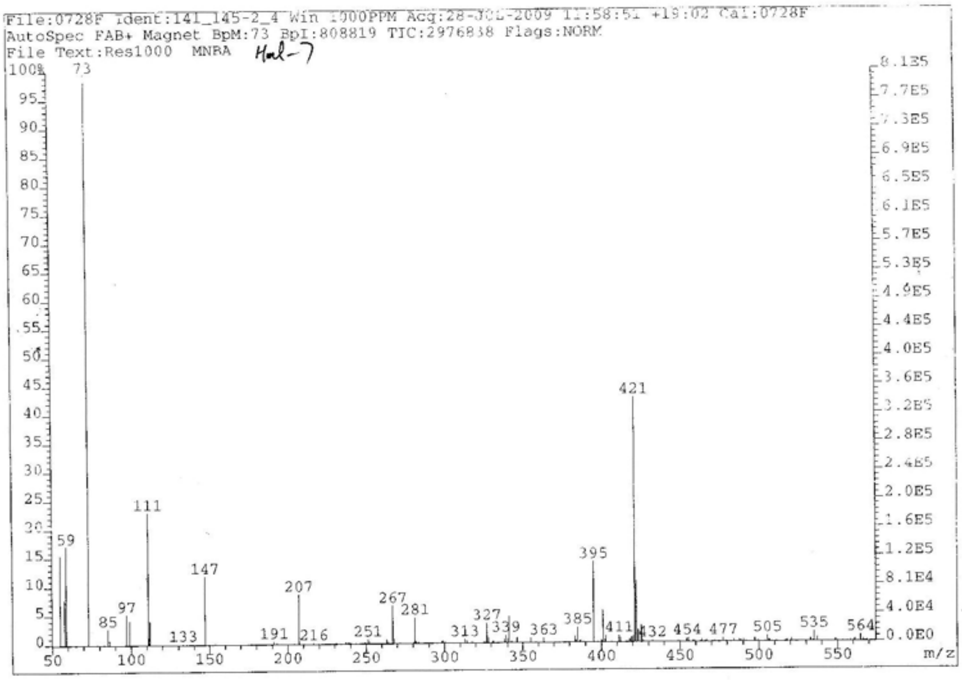

S27. UV spectrum of trigonoine C (**7**).

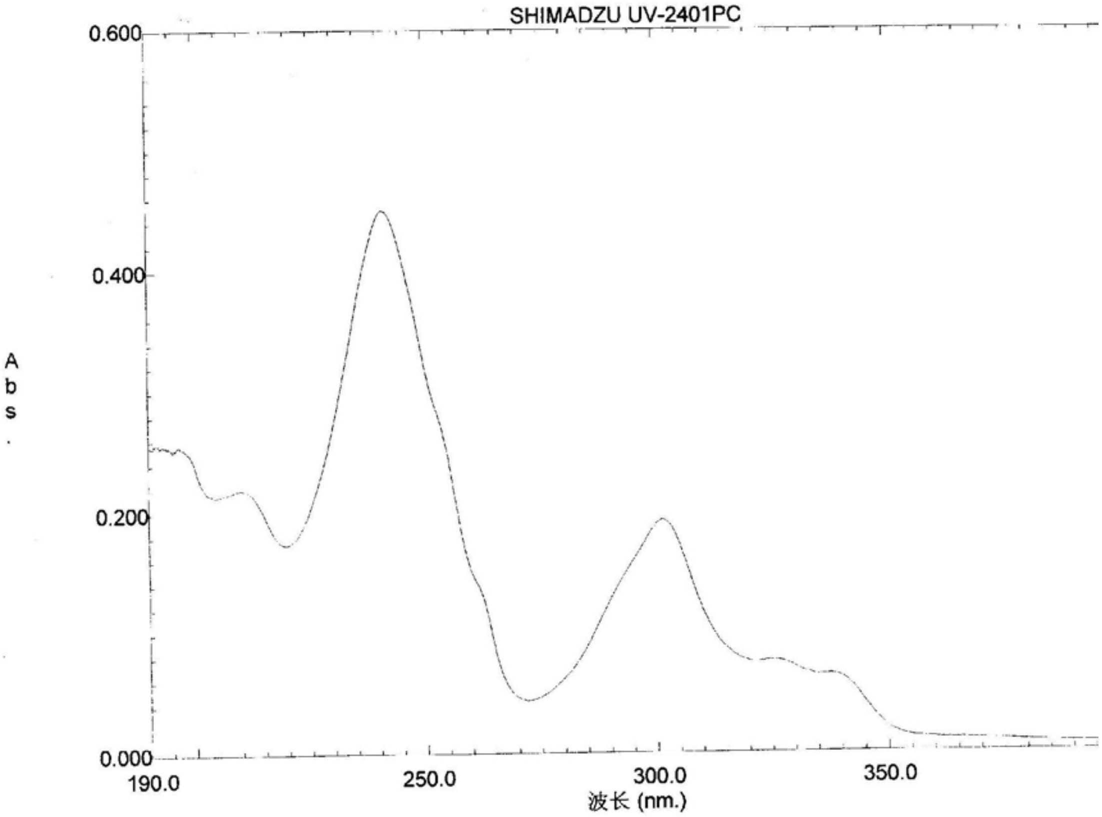

文件名: HML-7

HML-7 ———

样品浓度：0.0059毫克/毫升
溶剂：甲醇

创建于: 14:19 10-03-15
数据: 原始

测量模式: Abs.
扫描速度: 中速
狭缝: 2.0
采样间隔: 0.2

否.	波长 (nm.)	Abs.
1	337.60	0.0647
2	325.40	0.0761
3	301.60	0.1931
4	241.20	0.4504
5	209.80	0.2196
6	196.80	0.2556

S28. IR spectrum of trigonoine C (**7**).

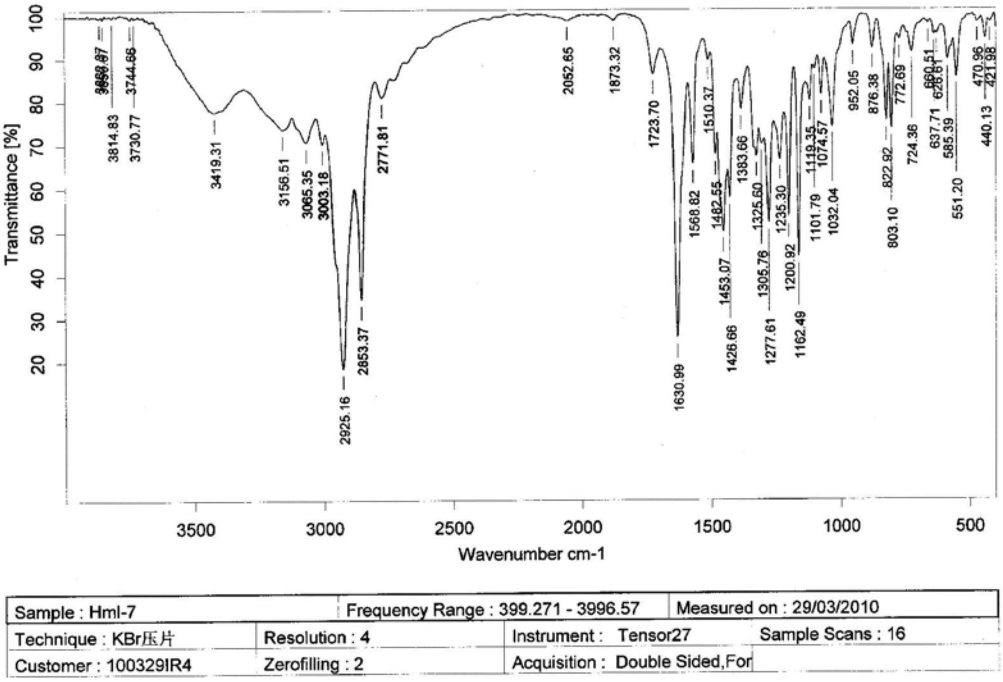

S29. ^{1}H NMR spectrum of trigonoine C (**7**).

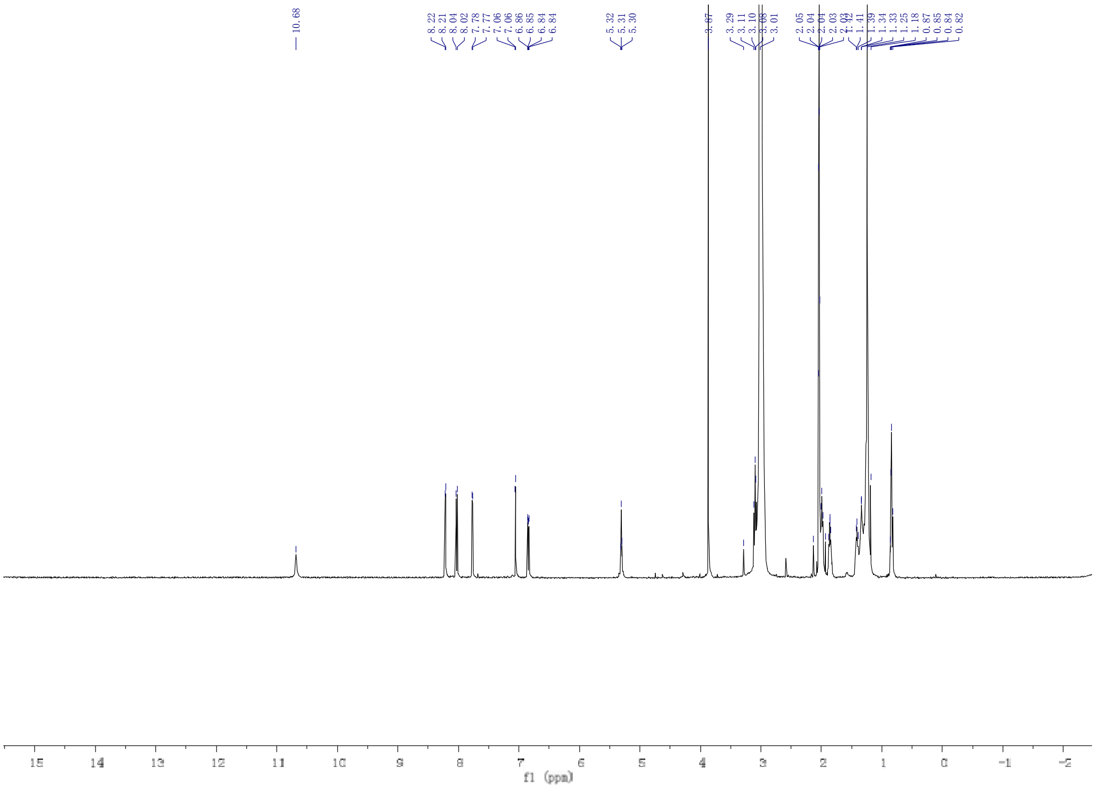

S30. ^{13}C NMR spectrum of trigonoine C (**7**).

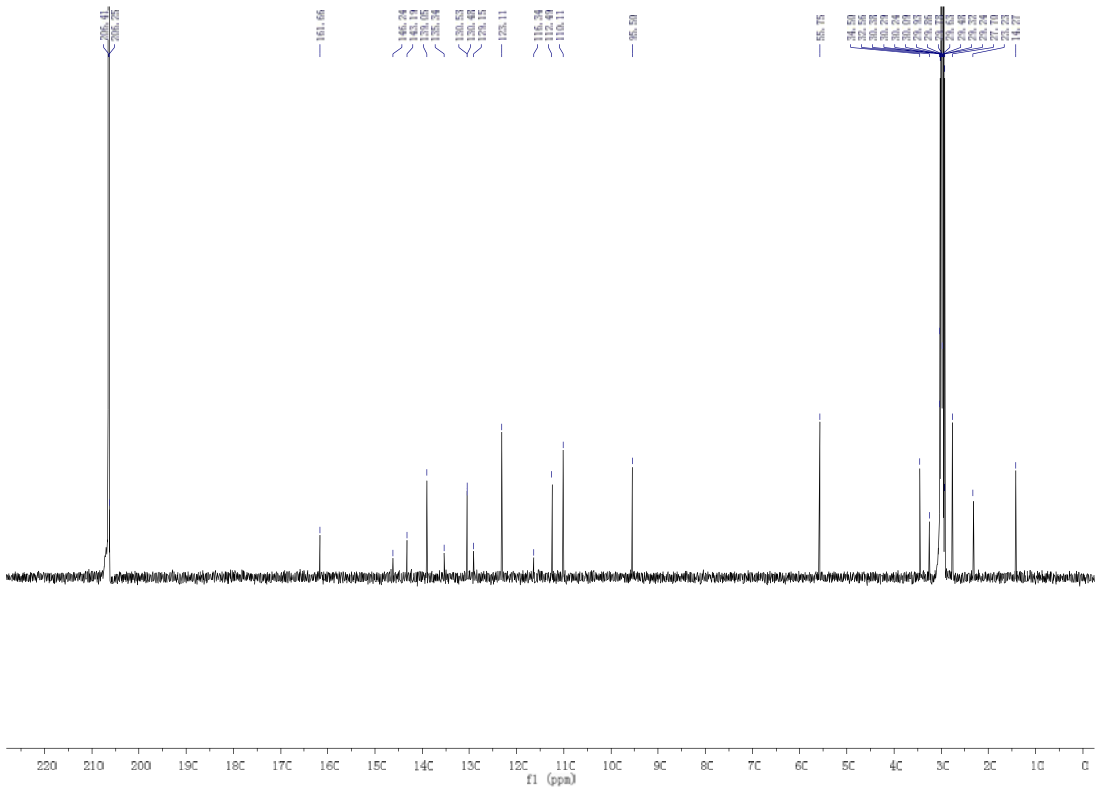

S31. HSQC spectrum of trigonoine C (**7**).

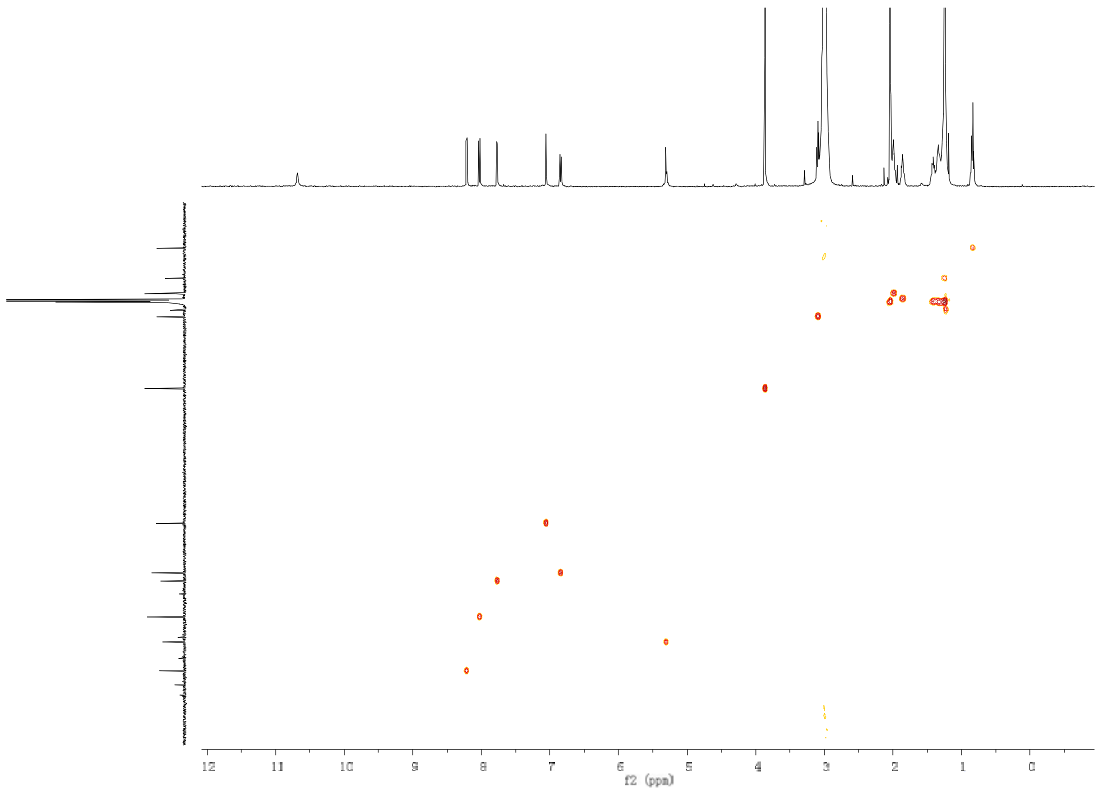

S32. ^{1}H-^{1}H COSY spectrum of trigonoine C (**7**).

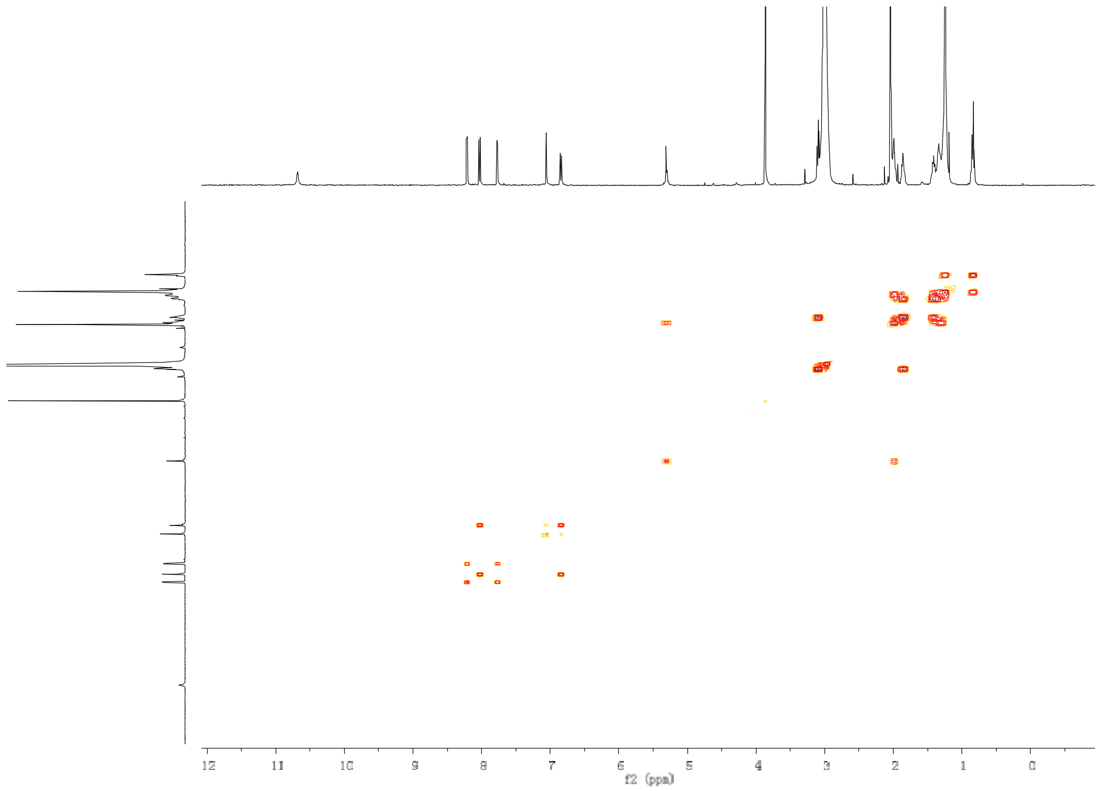

S33. HMBC spectrum of trigonoine C (**7**).

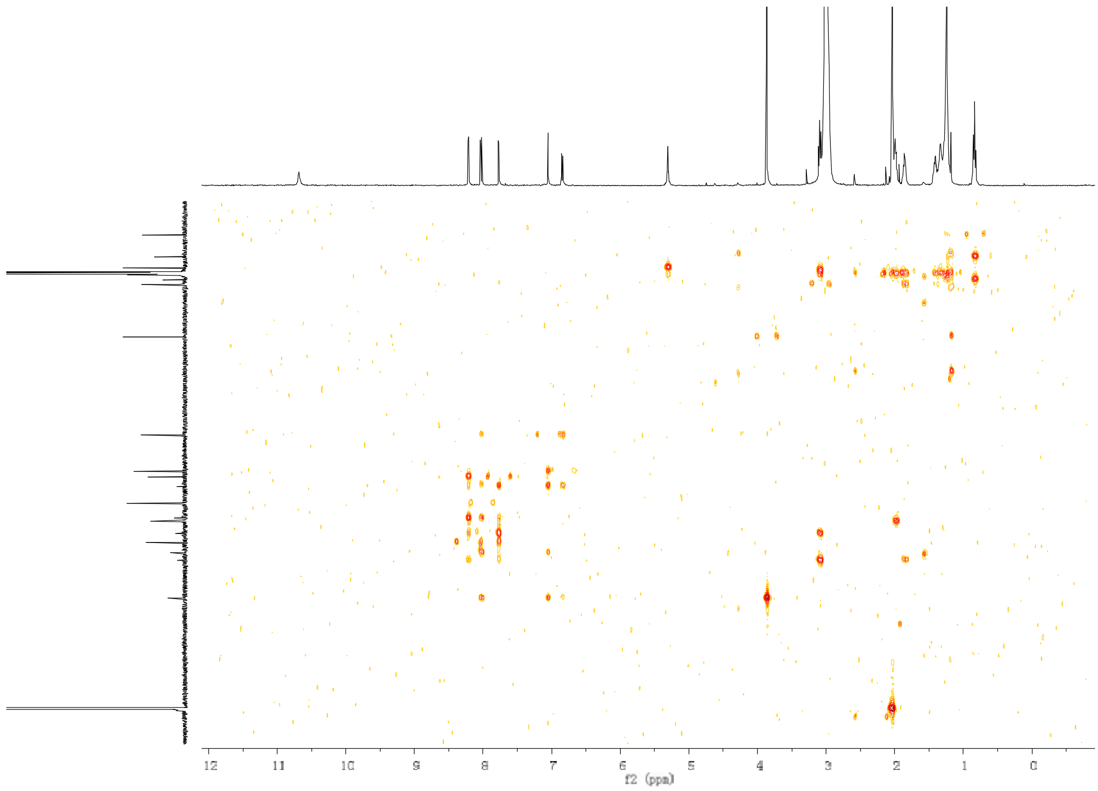

S34. LC-CD data analysis of trifiline A (**1**).

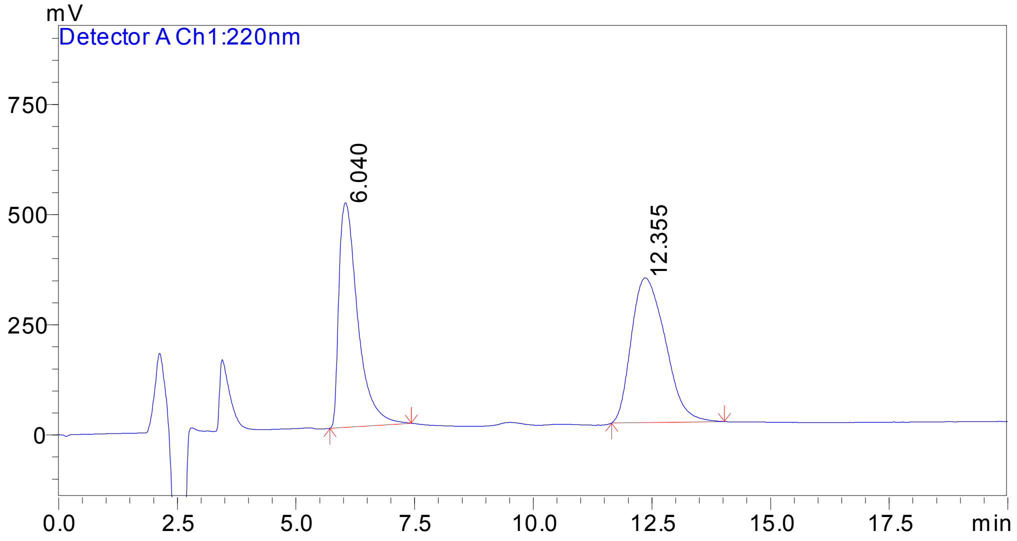

旋光谱图

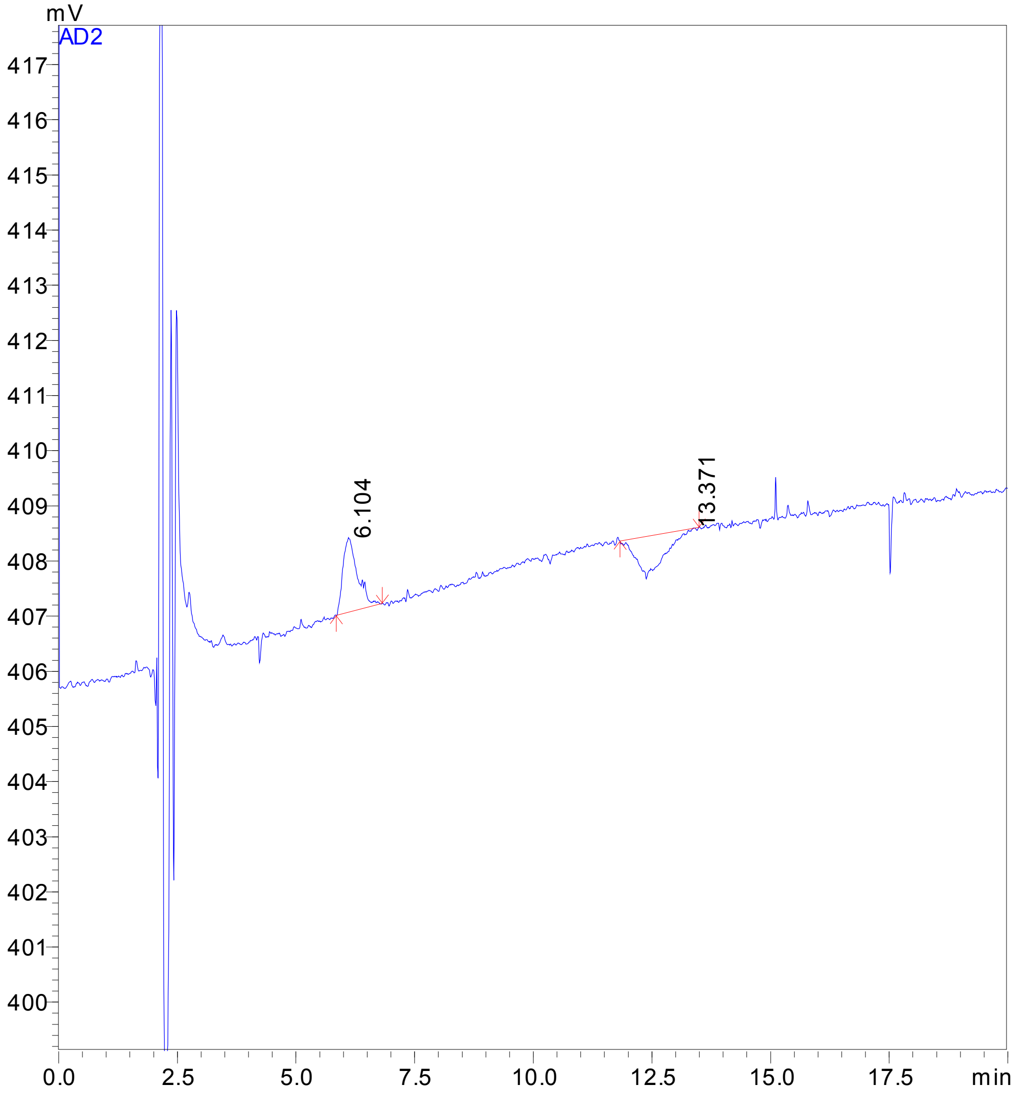

Supplement: Supplementary file 1 — Supplementary material, approximately 1.91 MB. [file 13659_2012_28_MOESM1_ESM.pdf]
